# Molecular and Pharmacogenetic Marker Evaluation in Relation to the Toxicity and Clinical Response of Acute Lymphoblastic Leukemia Treatment in Indian Children (MPGx-INDALL): Protocol for a Prospective Observational Cohort Study

**DOI:** 10.2196/79865

**Published:** 2026-03-17

**Authors:** Swetambri Sharma, Shuvadeep Ganguly, Kamali Murugadoss, Smita Kayal, Swaminathan Keerthivasagam, Jaikumar Ramamoorthy, Archna Singh, Deepam Pushpam, Jayanthi Mathaiyan, Yvonne Gloor, Frederic Baleydier, Marc Ansari, Sameer Bakhshi, Biswajit Dubashi, Chakradhara Rao S Uppugunduri

**Affiliations:** 1 Department of Medical Oncology, All India Institute of Medical Sciences, New Delhi, India; 2 Department of Medical Oncology, Jawaharlal Institute of Postgraduate Medical Education and Research, Dhanvantri Nagar, Puducherry, 605006, India, 91 7675935408; 3 Department of Pediatrics, Jawaharlal Institute of Post Graduate Medical Education and Research, , Puducherry, India; 4 Department of Biochemistry, All India Institute of Medical Sciences, New Delhi, India; 5 Department of Pharmacology, Jawaharlal Institute of Post Graduate Medical Education and Research, Puducherry, India; 6 Department of Pediatrics, Gynecology and Obstetrics, CANSEARCH Research Platform in Pediatric Oncology and Haematology, University of Geneva, Geneva, Switzerland

**Keywords:** pharmacogenetics, germline variants, somatic variants, toxicity, quality of life, treatment-related toxicity, ICiCLe, acute lymphoblastic leukemia, pediatric ALL, sequencing

## Abstract

**Background:**

Understanding interindividual variability in treatment response and toxicity is essential for optimizing outcomes in pediatric acute lymphoblastic leukemia (ALL). Molecular and pharmacogenetic markers hold promise in predicting treatment efficacy and adverse effects, particularly in genetically diverse populations. This protocol outlines the methodology for a prospective, nonrandomized observational cohort designed to evaluate molecular and pharmacogenetic factors associated with treatment response and toxicity in Indian children diagnosed with ALL.

**Objective:**

The primary objective is to identify genetic markers associated with treatment-related toxicity and therapeutic response. Secondary objectives include evaluating associations between the occurrence of early toxicities and quality of life during active ALL treatment, specific pharmacogenetic variants, and survival outcomes along with generating data to support the future implementation of personalized treatment strategies in Indian children with ALL.

**Methods:**

In this prospective, observational cohort, 556 children (≤18 years of age) with newly diagnosed ALL treated under the Indian Childhood Collaborative Leukemia–Acute Lymphoblastic Leukemia 2014 (ICiCLe-ALL-14) protocol at two Indian centers will be enrolled, aiming for a minimum of 500 evaluable children. Eligible participants will be enrolled prior to the initiation of chemotherapy and followed longitudinally throughout treatment. Clinical and laboratory data (demographics, nutritional assessment, quality of life, comorbidities, treatment regimen, toxicity graded by Common Terminology Criteria for Adverse Events v5.0, remission status, and survival) will be collected at predefined intervals up to day 100 of the maintenance phase. Germline and somatic DNA will be sampled at diagnosis and remission. The first phase will use whole-exome sequencing to discover candidate variants by implementing a candidate gene prioritization strategy. The second phase will genotype the top candidates in the full cohort using array technology. Associations with early treatment–related toxicities, steroid response, and survival will be tested by multivariable regression and Cox models. A machine learning approach with pharmacogenetic predictors as classifiers will be implemented further with cross-validation and sensitivity analyses.

**Results:**

Ethical committees approved the protocol version 1.0 in 2020: IEC-1167/06.11.2020 (All India Institute of Medical Sciences, New Delhi), JIP/IEC/2020/201 (Jawaharlal Institute of Postgraduate Medical Education and Research, Puducherry), and AO_2021-00048 (UNIGE, Geneva). Funding was received from Swiss National Science Foundation, Switzerland; Department of Biotechnology, India; and CANSEARCH Foundation, Switzerland. Recruitment began in December 2022 and is likely to conclude by 2027. A comprehensive analysis of the complete study cohort is anticipated to be completed by 2027.

**Conclusions:**

The MPGx-INDALL (Molecular and Pharmacogenetic Marker Evaluation in Relation to the Toxicity and Clinical Response of Acute Lymphoblastic Leukemia Treatment in Indian Children) study will generate actionable insights for individualized ALL therapy in India via systematically evaluating germline and somatic markers in a large ethnically distinct cohort.

## Introduction

### Background

Acute lymphoblastic leukemia (ALL) is the most common childhood malignancy accounting for about 25% of all cancers in children aged 2‐15 years [1]. Globally, the odds of surviving ALL have improved dramatically, with 5-year overall survival (OS) reaching up to 90% in high-income countries (HICs) [2,3]. The proportion of patient deaths due to treatment-related toxicity (TRT) during treatment remains significant, ranging from 2% to 24% [4]. There is clearly a survival gap between low-income countries and HICs. Over recent years, the treatment protocols in ALL treatment have been focused on minimizing TRT and long-term adverse effects while preserving favorable survival outcomes. The protocols developed by BFM (Berlin-Frankfurt-Münster), the Children’s Oncology Group, United Kingdom ALL group, and the French protocol for the treatment of ALL in children and adolescents are widely used in HICs [5].

Most of the oncology centers in India recently implemented these protocols, or modified versions of them, for the treatment of childhood ALL, especially the more recently implemented Indian Childhood Collaborative Leukemia–Acute Lymphoblastic Leukemia 2014 (ICiCLe-ALL-14) protocol [6-11]. The ICiCLe-ALL-14 protocol study on the Indian pediatric population reported a 4-year OS rate at 74%, and the event-free survival (EFS) rate was 62% [12]. Risk stratification clearly marked differences in the outcomes as well, with 4-year EFS and OS at 76% (95% CI 72%‐79%) and 88% (95% CI 85%‐90%) in the standard risk group, 70% (95% CI 66%‐74%) and 80% (95% CI 77%‐83%) in the intermediate risk group, 61% (95% CI 51%‐64%) and 73% (95% CI 70%‐76%) in the high risk groups, and 69% (95% CI 62%‐75%) and 77% (95% CI 70%‐83%) in T-ALL cases [12]. The cumulative incidence of treatment deaths and relapses at centers varied from 2% (95% CI 1%‐5%) to 13% (95% CI 10%‐17%; *P*≤.001) and 21% (95% CI 17%-26%) to 45% (95% CI 39%‐51%), respectively. About 39% of the relapsed cases were early relapses, and steroid poor response indicated worse EFS in univariate analyses having 53% of EFS in poor responders [12]. These results indicate addressing the issue of improving survival rates, but when it comes to the prevention of toxicities, we have no prospectively collected data from this geographical region. Further, delayed presentation at diagnosis, treatment abandonment [13-15], treatment-associated infections, adverse effects [16,17], higher steroid nonresponse rates (up to 18%-40%) [6,13], and higher incidence of relapse simultaneously account for the death of children with ALL undergoing treatment [14,18]. ALL is a genetically heterogeneous disease. Specific genetic variations have been identified as biomarkers to predict the toxicity and efficacy of drug therapies used to manage ALL [19], for example, mutations in the IL7R signaling components *JAK1* and *KRAS* with steroid resistance [20]. However, baseline prognostic stratification using genetic screening and risk-adapted protocols is yet to be uniformly accessible across low-resource settings and even in high-resource settings. The use of pharmacogenetic markers to guide the dosing of chemotherapeutic drugs is also nonexistent in low or low-middle income settings. Geographic and ethnic variations in childhood cancers [21] and variations in supportive care therapy further increase the need for population-specific, preemptive markers for optimized treatment management.

Indian children may harbor distinct genetic mutations [20] and risk profiles not well captured in Western studies. As Gogoi et al [12] emphasize, scaling up genetic profiling is both significant and transformative. Incorporating genetic testing into the management of childhood ALL—especially in India’s diverse and resource-limited setting—might help in the reduction of treatment-related mortality. Building on these insights, we would like to use this opportunity to combine this current standardized treatment protocol and the unique ethnic population of India, which also has high rates of inbreeding practices and consanguinity in certain parts of the country [22]. This prospective observational cohort study aims to evaluate the association of genetic variants (both germline and somatic) with TRTs, steroid response, and clinical outcomes among patients with childhood ALL being treated with the ICiCLe protocol.

### Objectives

The primary objectives of the study are as follows:

1. To study the associations of static germline genetic variants with early chemotherapy–related toxicities (treatment-related toxicities) in children with ALL undergoing the ICiCLe treatment protocol.
2. To investigate the somatic and germline genetic markers associated with the efficacy and toxicity of glucocorticoid response, respectively.
3. To biobank biological samples and clinical data for future association analyses to develop biomarkers predicting the efficacy and toxicity of the treatment protocol.

The secondary objectives of the study are as follows:

1. To study the impact of the occurrence of early toxicities on quality of life (QoL) during active ALL treatment (as estimated by PedsQL Cancer module tool).
2. To evaluate the association of genetic variants (somatic and germline) with OS, nonrelapse mortality, relapse-free survival (RFS), and EFS.

## Methods

### Study Design

**Figure 1.**
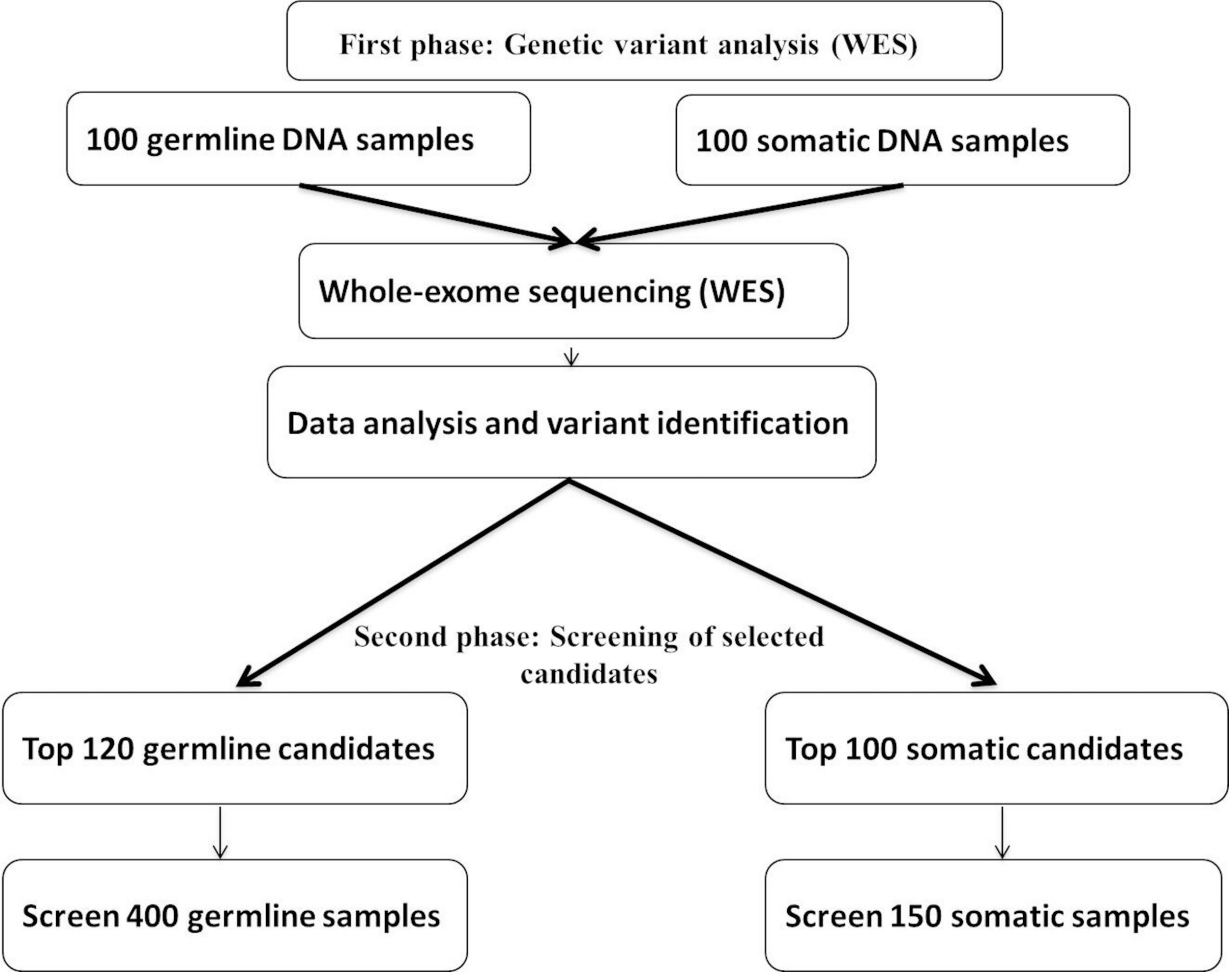
Study flow.

The proposed study is a prospective observational multicentric cohort study, where patient recruitment and clinical follow-up are being done at Regional Cancer Centre in Jawaharlal Institute of Post-Graduate Medical Education and Research, Puducherry in south India and at Dr. B.R.A. Institute Rotary Cancer Hospital, All India Institute of Medical Sciences, New Delhi in north India. The study is being carried out in 2 phases. The first phase includes the identification of germline genetic variants associated with TRTs and somatic mutations with that of steroid response using whole-exome sequencing (WES; n=100) for selecting candidate gene variants. WES combined with array technologies can shed light on many important coding region and regulatory region variants of interest. Through this exercise, we may also be able to identify any novel variants at a reasonable frequency in this ethnically distinct population. Further, selected variants from the first phase analysis will be genotyped in the rest of the cohort (n=400). Final association analyses will be performed using the entire cohort. First phase analyses will be conducted at the CANSEARCH Research platform of pediatric oncology and hematology, University of Geneva, Switzerland. Second phase analyses will be carried out using array technology at the two centers in India in collaboration with the University of Geneva, Switzerland. The study flow, including various phases, is shown in Figure 1.

### Patient Recruitment

Consecutive patients of pediatric ALL presenting to any of the treatment centers and being treated with the ICiCLe-ALL-14 protocol will be screened for the study.

The inclusion criteria of the study include patients (1) that are older than 1 year and younger than 18 years at enrollment; (2) that are previously untreated except for patients who received treatment up to 7 days of only steroid with documented baseline steroid dose details; (3) with an ALL diagnosis confirmed by morphology and immunophenotyping by flow cytometry; (4) with self-declared Indian origins; (5) who fulfill ICiCLe treatment protocol inclusion criteria and receive treatment as per the protocol; and (6) who provide written informed consent to participate in the study, signed by the participant, parent, or guardian. The exclusion criteria of the study include (1) previously treated patients and (2) patients with Down syndrome.

### Biological Sampling

The schedule of biological sampling planned for this study is mentioned in Multimedia Appendix 1. In brief, saliva or buccal swabs at the time of diagnosis or saliva or buccal swabs or blood at complete remission will be collected for germline DNA. For somatic DNA extraction, bone marrow samples will be collected at the time of diagnosis and at relapse (if any). In children with an inadequate amount of bone marrow, a peripheral blood sample will be considered for somatic DNA extraction, provided the percentage of blasts in peripheral blood is 80% or above. All saliva or buccal swabs and white blood cell pellets obtained from blood or bone marrow samples will be stored at 4 and −80 °C, respectively, until extraction. Plasma samples (4 mL whole-blood samples) will also be collected at the time of diagnosis (for drug level measurements), at remission, and at relapse (if any). Plasma separation will be performed immediately and stored at −80 °C. All biological sample aliquots remaining after addressing the study objectives will be stored in biobanks at respective centers for future investigations. Plasma or whole-blood samples will be collected at predefined time points. The details of the timelines for sample collection to estimate drug levels and biobanking are given in Multimedia Appendix 2.

### DNA Preparation Protocol, Samples Storage, and Shipment

DNA extraction will be performed in batches using QIAamp DNA Mini Kit. DNA quality and quantity will be determined using Nanodrop 260/280 ratio and Qubit [1] (Qubit 4 Fluorometer by Thermo Fisher Scientific). Integrity will be measured using Tapestation, with genomic quality number values greater than 7.5 being used for phase 1 analyses. An aliquot of 1 µg DNA will be shared with the Geneva team for phase 1 sequencing analyses and storage in the BaHOP (Biobank of the Pediatric Hematology and Oncology Unit, IRB number: PB_2017‐00533). The remaining aliquots will be stored in their respective biobanks in properly labeled vials at –80 °C temperature. DNA QC thresholds were as follows: minimum A260/280 ratio of 1.8‐2.0, Qubit-measured concentration 20 ng/µL or higher, and Tapestation genomic quality number less than 7.5 for phase 1 sequencing. At least 1 µg of germline DNA and 1 µg of somatic DNA per sample are required for shipment, with 2 or more additional aliquots stored at –80 °C at the center’s biobank for future investigations. DNA samples will be shipped on dry ice, with a maximum acceptable transit time of 48 hours and temperature logs maintained. For plasma sample separation, whole blood must be centrifuged within 30 minutes of collection, and separated plasma shall be aliquoted into prelabeled cryovials and immediately stored at −80 °C until shipment on dry ice. All the harmonized laboratory and sampling procedures have been finalized and are provided in the laboratory manual (Multimedia Appendix 2) to support consistent implementation across centers.

### Clinical Data Collection, Patient Follow-Up, and Recording of Outcomes

The baseline sociodemographic details, including socioeconomic status by the modified Kuppuswamy socioeconomic scale and clinical and laboratory details (immunophenotype, karyotyping, and molecular tests) during disease presentation, will be collected [23]. All patients will undergo risk stratification and receive treatment as per ICiCLe-ALL-14 protocol and use uniform case record forms implemented in REDCap (Research Electronic Data Capture). The data will be anonymized by each center before the sharing of the clinical data and genetic data with other parties and in the public domain.

Patients will be followed up at each outpatient visit and any unscheduled visit due to toxicity. At each visit, all the available details of drug dose, modifications, or omissions due to toxicities will be recorded. This will aid in assessing the intensities of the treatment in relation to the incidence of toxicities and other clinical outcomes. All drug-related toxicities as per common terminology criteria for adverse events (version 5.0) [24] occurring from the first day of induction until day 100 of maintenance therapy will be recorded. For objective 1 (primary end point analyses), a predefined set of clinically significant early toxicities will be evaluated, including grade 3 or higher hematological, hepatotoxicity, pancreatitis, neurotoxicity, and serious infections. These events are explicitly mapped to the case record forms to ensure harmonized data capture across study sites. Post day 100 of maintenance, the patient will continue to be monitored for long-term outcomes including relapses (if any), need for hematopoietic stem cell transplantation, and mortality.

The data on steroid response at the end of 7 days (1 week of steroid prophase), bone marrow morphological remission status, and minimal residual disease using flow cytometry status at the end of induction or consolidation (if applicable) will be systematically recorded and analyzed in relation to somatic genetic variants.

Other clinical outcomes to be collected are nonrelapse mortality (time from study enrollment to death due to cause other than relapse of disease), incidence of relapse of disease (ie, the duration between the day of complete remission and the day of occurrence of relapse), RFS (time from study enrollment to relapse for patients attaining complete remission), OS (the duration from enrollment in the study to death due to any cause), and EFS (from treatment initiation to the first induction failure, nonresponse, or the progression of the disease, death from any cause).

Additionally, QoL will be assessed by the interview of children and their parents or caregivers using the age-specific PedsQL Cancer Module v3.0 questionnaire [25]. QoL will be assessed longitudinally initially at baseline, then at end of induction, consolidation, interim maintenance, and at day 100 of maintenance phases of the ICiCLe-ALL-14 protocol. We have obtained a license to use the questionnaires (work order 312005), and translated versions are validated as per the guidelines of the Mapi research trust and are made available at the Mapi research trust. Nutritional status will be evaluated by anthropometric assessment at baseline and serially along with the recording of the implementation of any nutritional recommendations. The case record form template used for clinical data collection and XML files of the REDCap database template are available as MultimediaAppendices 3  4, respectively.

### Schedule of Patient Participation in the Study

The patient follow-up data will be collected for the study objectives from the day of induction treatment until day 100 in the maintenance phase of the ICiCLe treatment protocol for ALL. Each patient will be scheduled for data collection and laboratory tests as a part of routine clinical care for 37 to 42 weeks from the day of the initiation of the treatment to evaluate the primary objectives and one of the secondary objectives related to QoL. However, the survival outcomes would require having follow-up data up to 1 year post treatment. The details of the planned procedures for patients and timelines are shown in Multimedia Appendix 2.

### Detection and Analysis of Genetic Variants

#### First Phase Genetic Variant Analysis

##### Whole-Exome Sequencing

WES will be performed in 100 germline DNA samples and 100 DNA samples from leukemic cells (same patients). WES will use the TWIST Biosciences TWIST Comprehensive Exome workflow according to manufacturers’ protocols: (1) library preparation; (2) sequencing using an Illumina NovaSeq6000 system with a mean coverage of 70× for germline DNA samples and 150× for somatic DNA samples; and (3) raw data integration, storage, and analyses. The sequencing will be done at the Health 2030 Genome Center, Campus Biotech, Geneva, Switzerland, and the sequences will be stored on a secured server of the University of Geneva until analyses and at the corresponding centers until the end of the study and archival.

##### Genetic Data Analysis—First Phase

For genetic data analysis, whole exomes and flanking regions (n=100, paired somatic and germline DNA) captured will be mapped to the GRCh38 reference and the variants called using the DRAGEN v.4.0.3 analysis platform. Plink 1.9 will be used to perform the quality control of the resulting sequences including call rate, Hardy-Weinberg equilibrium, sex mismatch, relatedness, heterogeneity, and ethnicity. Quality control investigations on cryptic relatedness and concordance between the reported and genetic sex will be included for patient selection. Principal component analyses will be an integral part of the analyses due to the possibility of unique clusters. Thus, the population structure is also accounted for while performing association analyses. When it comes to pharmacogenetic association (objective 1), frequencies might be impacted by the population structure, but for somatic genetic association, we expect no impact of population structures.

We will perform association analysis via (1) a candidate gene approach with filtered variants or mutations to the selected genes using candidate prioritization strategies from a systematic review (PROSPERO CRD420251112557 and CRD42021229748) and candidates associated with phenotypic measures used for toxicity definitions or steroid sensitivity from public datasets, for example, UK Biobank (application 91515), and (2) a hypothesis-free, exome-wide, and association analysis. The predicted effects of missense variants on protein function will be assessed in silico using SIFT and PolyPhen2 incorporated into variant effect predictor tools [26]. Variant filtering will be performed based on the 1000 Genomes, UK Biobank, IndiGen variation [22], and the NHLBI GO Exome Sequencing projects [27]. Fisher exact test (allelic association) and the Cochran-Armitage test for trends will be implemented in PLINK [28] to search for associations between clinical outcomes and genetic variants. Association analysis for quantitative and binary data will be analyzed using general linearized models in PLINK, with a *P* value significance threshold of .05 after adjustments for multiple testing that will be performed using the Benjamini-Hochberg false discovery rate method [29]. Candidate prioritization strategies for phase 1 analyses will be communicated in a separate report. Phase 1 analyses will thus enable the creation of a leukemia sequencing database of a single ethnicity, along with its clinical and follow-up data, all obtained from a single study. This will serve as a valuable resource for researchers investigating questions related to ALL treatment in children.

### Second Phase Genotyping for Selected Candidates From Phase 1 Analysis

Somatic short variants will be called using GATK Mutect2 with matched tumor (bone marrow) and normal (buccal swab) samples. To control for germline mutations, we will provide a panel of normals generated from the buccal swab samples (GATK CreateSomaticPanelOfNormals) and use the gnomAD af-only resource (af-only-gnomad.vcf.gz; reference set of germline mutations) as our germline resource. Cross-sample contamination will be estimated (GATK GetPileupSummaries+CalculateContamination) and used during filtering. The resulting calls will be filtered and annotated for downstream analyses (GATK FilterMutectCalls) “purity thresholds.” Tumor purity and copy number will be inferred using PureCN, as well as computing cancer cell fraction for each somatic variant. Samples with estimated tumor purity *p* less than 20% will be excluded from primary discovery analyses (but retained in QC tables). This threshold may be refined after inspecting the purity distribution. The rationale behind choosing this threshold is as follows: for a heterozygous clonal mutation (c=1) in a diploid region, the expected variant allele frequency (VAF) of a clonal mutation would be *p*/2 (eg, when *p*=1.0, VAF=0.50, when *p*=0.20, VAF=0.10). A mutation present in only 10% of the tumor cells (subclonal, cancer cell fraction=0.1), and tumor purity *p*=0.20, would correspond to a VAF of 0.01, which corresponds to 1.5 alternate reads at 150×. This is below a reliable detection level that can be confounded with sequencing noise. Additionally, to reduce false positives from sequencing noise, we will require a minimum of 5 alternate reads for somatic calls to pass filters for downstream analyses.

Phase 2 analyses will include screening 400 germline DNA samples for approximate top candidates identified in phase 1’s germline sequencing association analysis with TRT. Somatic DNA analysis includes the screening of 150 samples from the top 100 candidates from phase 1 analysis. The final TRT association analyses will be conducted for 500 patients for the top candidates and for steroid response (steroid or prednisone response rate on day 8; good responders: peripheral blood blast count <1000/µL; poor responders: peripheral blast count ≥1000/µL) [7] in 250 patients before meta-analysis with phase 1 participants. Phase 2 genotyping will be realized by genotyping arrays. We further plan to develop a project for cost-effective screening for implementation in a clinic, for example, custom open array.

### Withdrawal and Discontinuation

The expected dropout rate is low for the entire period of the study. However, it is expected that approximately 5% of the recruited patients may discontinue or drop the study, and this will be managed by recruiting new patients into the study, if the data are not available for use to evaluate primary objectives.

### Statistics and Analyses Methodology

SPSS (version 25; IBM Corp) or R statistical software (packages: survival, cmprsk, glmnet, and mbmdr [30]) will be used for statistical analysis. The appropriate genetic models (dominant, recessive, or additive) for specific genotypes or haplotypes will subsequently be derived based on the results of those analyses. Nonparametric or chi-square tests will be used to test differences in sex, age, and other categorical variables between genotype groups. The allele genotype frequencies, Hardy-Weinberg equilibriums, and haplotypes will be analyzed using Plink v1.9, the Haploview software (Broad Institute), and PHASE [31]. The frequency of all incidents and toxic events will be compared between genotype or haplotype or demographic- and disease characteristic–based groups using chi-square tests. The cumulative incidences of clinical outcomes, such as TRTs, will be estimated in relation to genotype or haplotype groups using cumulative incidence curves (cmprsk package in R). Survival curves for OS, nonrelapse mortality, RFS, and EFS will be calculated using Kaplan-Meier estimations, and log-rank tests will be used to compare the differences between genotype groups in univariate analysis. Univariate Cox regression analysis will be used to estimate hazard ratios with 95% CI; multivariate Cox regression analysis will then be used to estimate the impact of genotypes or haplotypes on clinical outcomes in the presence of other covariates. The influence of multiple genes on clinical parameters will be assessed in gene-gene interaction epistasis models incorporating various genetic and clinical confounders using multivariate regression analysis and multifactor-dimensionality reduction analysis [32]. All primary genetic association models will be adjusted for a predefined set of covariates but not limited to age, sex, immunophenotype, baseline white blood cell count, measurable residual disease, risk category, nutritional status category, socioeconomic status (modified Kuppuswamy), and treatment center.

We will use appropriate statistical tests for less frequent outcomes, for example, Least Absolute Shrinkage and Selection Operator–penalized regression [33], thus avoiding the overfitting of the data and biasing results due to the collinearity of the factors included. Confounders, such as socioeconomic and nutritional status, will also be included in the analyses. Reduced QoL due to the occurrence of toxicity will also be evaluated using logistic regression (after defining the impaired scores for both physical and emotional components). PedsQL scores are collected longitudinally across treatment phases; hence, we will attempt mixed-effects modeling to appropriately account for within-patient correlation and temporal trends. The objective of the final joint analyses is to determine the sensitivity and specificity of gene variants combined with other patient-specific characteristics to predict the clinical outcomes. This will be analyzed by decision curve analysis using all the categorical variables (some continuous variables would be converted into categorical variables using the X-tile program from Rimm lab, Yale School of Medicine), including genotypes along with reclassification improvement and integrated discrimination improvement in R using the “rms” package. The concordance index would be used to test a new model in comparison to the previous model. The performance of this strategy would be compared with that of receiver operating characteristic curve analysis. We will be applying suitable models only after the exploration of the data based on the collinearity matrix of the variables. However, we hereby propose possibilities for the analyses a priori. Appropriate statistical tools will be implemented upon discussions with peers to derive meaningful conclusions from the data collected in secondary analyses.

Additionally, for multiplicity control, the 2-stage discovery-validation design incorporates explicit control for multiple comparisons. In phase 1 (WES discovery), variant associations will undergo Benjamini-Hochberg false discovery rate (FDR) control (target FDR=0.05) with the reporting of both *q*-values and unadjusted *p*-values. Only the top ≈120 predefined candidates meeting prespecified criteria (biological relevance, effect size, and FDR ranking) will advance to phase 2 validation. In phase 2, confirmation analyses will apply a Bonferroni-adjusted significance threshold (*α* = .05/number of candidate variants). Joint (meta-analytic) models combining phase 1 and phase 2 will apply the same validation-level α threshold. This strategy balances discovery sensitivity with stringent clinical-grade validation.

### Power Considerations for Primary Objectives

For germline pharmacogenetic variant associations, the sample size (n=500) was calculated using a power calculator for genome-wide association studies using a 2-stage design [34]. In this 2-stage design, 20% of the patients are genotyped for all the candidate markers, and then the remaining 80% are genotyped for the top 120 candidates (0.02% of markers analyzed in phase 1). This design has sufficient power for the phase 1 analyses: replicating and joint analyses. Criteria used for estimation are 5% to 15% minor allele frequency; dominant mode of inheritance; α value of .00001 (0.05/5000; 2-sided); power (1 – β) of 80%; early toxicities prevalence of 40%; and relative risk of 3.5 to estimate for the gene variant or variants. We used a stringent α value of .00001 (instead of .0004), that is, adequate for joint analysis (.05/120 = .0004) as required when developing models for clinical use. We acknowledge that this approach may lack smaller but clinically important associations in resource-limited settings. This design reduces the total amount of genotyping required by 80% (and thus associated costs), and there is a 90.1% probability that the associated markers will be followed in stage 2 analysis. Considering 5% of patients with loss to follow-up and an additional 5% of samples being not analyzable due to laboratory issues, a total of 556 patients need to be recruited to get 500 evaluable patients for the primary objective 1. For secondary outcomes (OS, EFS, QoL, and steroid response), the power considerations are detailed in Multimedia Appendix 5.

### Handling of Missing Data

This study expects to have minimal missing data, as a dry run for collecting data will be implemented, and documented issues will be fixed before initiating the recruitment of the patients. Moreover, the study includes participants who are under routine clinical care settings (in-patient settings and out-patient settings) for about 2.5 years, and our primary objective’s data collection is limited to a maximum of 40 weeks; hence, we foresee dropouts (5%) from the study. Most importantly, the social worker coordinating the ALL treatment at both centers will ensure that patients receive this standard treatment. For secondary survival outcomes, we will collect the data retrospectively through forms that are currently filled out by the participating centers. The few dropouts that may occur shall be replaced by the recruitment of new participants. Patients whose germline samples could not be collected or analyzed will be excluded from the study and will not be analyzed. Furthermore, patients requesting the withdrawal of their informed consent will not be analyzed by the end of the study.

### Ethical Considerations

The ethical approval to conduct the study was obtained from the Institute Ethics Committee, All India Institute of Medical Sciences, New Delhi, India (IEC-1167/06.11.2020); Jawaharlal Institute of Postgraduate Medical Education and Research, Puducherry (JIP/IEC/2020/201); and a positive stance from the ethics committee of Canton of Geneva (AO_2021‐00048). All study procedures will be conducted in accordance with the principles of the Helsinki Declaration and in accordance with national and local regulations. All consecutive eligible patients will be recruited in the study after informed consent from a legal guardian and patient assent (if applicable). The participant or their legal representatives may withdraw consent to participate in the study at any time without justification, and without affecting the quality of routine standard medical care. For any withdrawal of consent, the data collected prior to that point will be used unless the participant or his or her legal representative does not wish to do so in writing.

## Results

As this is a protocol, no clinical or genetic results are presented here. This section summarizes the current study status. Ethical approval has been obtained from all participating centers, and recruitment began in December 2022. A total of 346 participants were recruited for the study, and recruitment is ongoing. Phase 1 exome sequencing for both germline and somatic DNA has been completed. As per the most recent internal review, recruitment is projected to conclude by 2027. Data cleaning, harmonization, and preliminary genetic analyses for phase 1 are planned to begin in early 2026, with full cohort analyses expected in 2027.

## Discussion

### Principal Findings

To date, there have been no comprehensive studies for the treatment of childhood ALL across India’s unique ethnic population in a standardized treatment setting with predefined methodology. We would like to use this opportunity to initiate this comprehensive project to serve as a benchmark pharmacogenetic study and to provide valuable resources (biological material and clinical data) for future investigations aimed at personalizing ALL treatment in Indian children. The novelty of the study lies in the fact that the 2-stage design with candidate prioritization strategies is implemented in a prospective cohort that exclusively covers the ICiCLe protocol that is uniformly used to treat patients with pediatric ALL across the Indian population. Moreover, harmonized treatment protocols in India have increasingly demonstrated benefits to survival progressively. This study design will enable us to identify both common and specific variants as markers of TRTs and clinical outcomes of ALL treatment in Indian children.

The study’s results will be useful in the future to apply therapies to children and adolescents from India and elsewhere being treated for ALL. The concept of avoiding lost-to-follow-up patients by preventing the development of toxicities through personalized dosing is innovative. Thus, identifying such patients earlier using genetic markers would enable the physician to plan a proper management of their therapies well in advance, which is advantageous in limited-resource settings. Glucocorticoids are the principal components of pediatric ALL therapy protocols, including ICiCLe. It has been established through previous studies that there is interindividual variability in the treatment response to glucocorticoids as well as in susceptibilities to their toxicity [19,20]. Steroid nonresponders are usually at a higher risk of treatment failure, but response assessment is done after 8 days of treatment in the current treatment regimen. Therefore, with the early identification of responders, this study will help in reducing deaths related to steroid nonresponders. As the treatment protocol involves several chemotherapeutic drugs, there is an inadvertent need to identify genetic markers involved in drug metabolization, transport, and pharmacodynamics. So far, studies have been focused on selecting single genetic markers, but there has been emerging evidence suggesting associations between single-nucleotide polymorphisms and the development of toxicity or resistance or relapse to ALL therapies. Various single-nucleotide polymorphisms (target genes or metabolic enzymes) have been identified that are associated with increased susceptibility to drugs used in treatments, such as thiopurine methyltransferase, nudix hydrolase 15 for 6-mercaptopurine, methylenetetrahydrofolate reductase for methotrexate, and asparagine synthetase for L-asparaginase. This study will assess the relevance of these findings in addition to shedding light on novel variants, leading to a more comprehensive genetic profile for each patient that may aid in developing precision medicine strategies tailored specifically for the Indian population.

Therefore, it is our expectation that this study will provide direction on the utility of using genetic biomarkers for patient stratification. If the proposed genetic stratification criteria could be clinically justified, it would be possible to involve preemptive genetic testing in routine clinical practice. As the study spans 2 centers and 2 laboratory environments, we prospectively established harmonized standard operating procedures and predetermined covariate sets. Batch-effect control measures, including technical replicates and platform-specific QC pipelines, have been incorporated to minimize systematic bias. The study will include whole-exome discovery with downstream genotyping; integration of clinical, QoL, and biological data; and biobanking for future work. This study design is not devoid of any limitations; for example, the design includes the evaluation of the most frequent variations and lacks power to quantify the effect of rare variants. The 2-phase design of the study optimizes the costs of genotyping but compromises study power to quantify the effect of low-frequency variations that may have impact together in a gene-gene interaction model. Moreover, the sample size of the study is calculated considering objective 1, that is, germline genetic predisposition. Within this study, we do not plan to collect late toxicities due to follow-up limited to day 100 of maintenance for primary objectives and reduced power for detecting modest genetic effects. However, patients will be followed up to obtain the retrospective data collection on toxicities and other clinical outcomes to assess long-term clinical outcomes in relation to germline and somatic genetic variants. Another major limitation includes the inclusion of patients from 2 geographical locations ie, North and South India, that are distinct in terms of ethnic origins. However, there is no clear distinction that was observed in terms of pharmacogene variant distribution between these 2 distinct groups [14]. This limitation, indeed, is an advantage to identify the variants that may have clinical utility for both populations, so that countrywide policies can be made efficient for translating and implementing the findings in clinics. Another limitation is the long recruitment time that may have an impact on the institute’s policies and practices in treating the disease using the ICiCLe protocol. This can be resolved by considering treatment year as a confounder in the association analyses. This protocol study is reported in accordance with the SPIROS (Standardized Protocol Items Recommendations for Observational Studies) and STROBE (Strengthening the Reporting of Observational Studies in Epidemiology) guidelines for observational studies and the TRIPOD-AI (Transparent Reporting of a Multivariable Prediction Model for Individual Prognosis or Diagnosis plus Artificial Intelligence) guideline for studies involving the development of machine learning–based prediction models.

### Conclusion

To conclude, this study will prospectively evaluate the association of germline genetic variants with TRT and clinical outcomes in patients with pediatric ALL of Indian origin. The study will also explore somatic variant associations with clinical outcomes in the background of socioeconomic and nutritional status. In addition, the impact of toxicity occurrence on QoL will also be assessed as well, along with storing biological material and clinical data for future studies.

## Supplementary material

10.2196/79865 Multimedia Appendix 1 Details of biological sampling.

10.2196/79865 Multimedia Appendix 2 Laboratory manual.

10.2196/79865 Multimedia Appendix 3 MPX-IND ALL Case Record Form Version 1.3.

10.2196/79865 Multimedia Appendix 4 Electronic case record form template (REDCap).

10.2196/79865 Multimedia Appendix 5 Objectives 2 and 3.

## Abbreviations

ALL: acute lymphoblastic leukemia
EFS: event-free survival
FDR: false discovery rate
HIC: high-income country
ICiCLe-ALL-14: Indian Childhood Collaborative Leukemia—Acute Lymphoblastic Leukemia 2014
MPGx-INDALL: Molecular and Pharmacogenetic Marker Evaluation in Relation to the Toxicity and Clinical Response of Acute Lymphoblastic Leukemia Treatment in Indian Children
OS: overall survival
PIROS: Standardized Protocol Items Recommendations for Observational Studies
QoL: quality of life
REDCap: Research Electronic Data Capture
RFS: relapse-free survival
STROBE: Strengthening the Reporting of Observational Studies in Epidemiology
TRIPOD-AI: Transparent Reporting of a Multivariable Prediction Model for Individual Prognosis or Diagnosis plus Artificial Intelligence
TRT: treatment-related toxicity
VAF: variant allele frequency
WES: whole-exome sequencing
