# Supplementary material for: Molecular and Pharmacogenetic Marker Evaluation in Relation to the Toxicity and Clinical Response of Acute Lymphoblastic Leukemia Treatment in Indian Children (MPGx-INDALL): Protocol for a Prospective Observational Cohort Study
Source: JMIR Res Protoc. 2026 Mar 17;15:e79865. doi: 10.2196/79865 (PMC12994881; doi:10.2196/79865)
Supplement: Multimedia Appendix 1 [file resprot-v15-e79865-s001.docx]

***Supplementary material S1***

Details of biological sampling

| Sample collection | At diagnosis | At the end of inductionor when remission is complete | At relapse |
| --- | --- | --- | --- |
| Mandatory | DNA from a Bone Marrow (BMD) sample & BM cells for extraction.  If BM cannot be obtained, a peripheral blood sample (provided peripheral blood blast percentage ≥80%) will be used (OGD).1  Buccal swab or saliva for nucleic acid extraction (GLD)2  *OGD sample will not be collected for patients:  - BM not available and peripheral blood blasts ≤80%  - Patient has received steroids up to 7 days before study inclusion | A. A peripheral blood sample taken at complete remission (GLD) which is a preferred sample OR collect buccal swab or saliva for nucleic acid extraction (GLD) 2;  *Patients who do not achieve remission (refractory disease) or remains MRD positive, peripheral blood sample for GLD will not be collected. | A. DNA from a Bone Marrow (BMD) sample and BM cells for extraction.  If BM cannot be obtained, a peripheral blood sample (provided peripheral blood blast percentage ≥80%) will be used |
| Optional | Plasma sample (2 mL)-proteomic analysis and miRNA analysis (PD). 3 | Plasma sample (2 mL)-proteomic analysis and miRNA analysis (PI) | Plasma sample (2 mL)-proteomic analysis and miRNA analysis (PR) |
