# Supplementary material for: Molecular and Pharmacogenetic Marker Evaluation in Relation to the Toxicity and Clinical Response of Acute Lymphoblastic Leukemia Treatment in Indian Children (MPGx-INDALL): Protocol for a Prospective Observational Cohort Study
Source: JMIR Res Protoc. 2026 Mar 17;15:e79865. doi: 10.2196/79865 (PMC12994881; doi:10.2196/79865)
Supplement: Multimedia Appendix 2 [file resprot-v15-e79865-s002.pdf]

LABORATORY MANUAL

Molecular and Pharmacogenetic marker evaluation in relation to the toxicity and clinical response of acute lymphoblastic leukemia treatment in Indian children

(MPGx-INDALL)

ClinicalTrials.gov Identifier: NCT05512169

Version Control Log

Version	Date	Editor
Version 2.0	13-08-2025	Ms. Kamali M
Version 1.9	18-09-2024	Dr. Govindan K
Version 1.8	03-04-2024	Dr. Govindan K
Version 1.7	11-12-2023	Ms. Kamali M
Version 1.6	08-12-2023	Ms. Swetambri S
Version 1.5	17-11-2023	As a team
Version 1.4	07-08-2023	As a team
Version 1.3	28-02-2023	Ms. Kamali M
Version 1.2	17-02-2023	Ms. Swetambri S
Version 1.1	28-11-2022	Ms. Swetambri S

Change Log

Version 2.0:	Added version control log, change log, Annexure-H & I, Catalog no's for reagents, uniformity in representation of units, refined page no's, added ICH -GCP link, checklist for sample shipments etc.
Version 1.9:	Corrected for Alignments and Grammer errors
Version 1.8:	Details of SRF change in JIPMER center updated
Version 1.7:	Update on note to utilizing the leftover volume of samples to be used for extraction in different columns.
Version 1.6:	Added steps of buccal swab collection
Version 1.5:	Updated details drug levels laboratory
Version 1.4:	Refinements of the second drafts
Version 1.3:	Added newly joined staff details and labels stickers
Version 1.2:	Discussion and update
Version 1.1:	Initial draft

**Table of contents**	**Page No**
List of Abbreviations	04
Contact information	05
Introduction	06
Section-1: Preparation for sample collection	07
Section-2: Collection of blood samples	10
Section-3: DNA Extraction & Quality check	19
Section-4: Courier of TDM samples	21
Section-5: Sample Preparation and Packaging	21
Section-6: Instructions of courier	22
Appendix -A1: Flowchart for Specimen Collection time points, sample type and storage	23
Appendix A2: Consumable list - a) Blood and b) Bone marrow collection and processing	24
Appendix B: Schedule of assessments	25
Appendix C: Study specific sampling details: Real time shipment to courier (internal within the institute or to analytical laboratory or pathology laboratory)	27
Appendix D: Summary of storage, processing and transport logistics.	29
Appendix E: Routine Laboratory Testing	30
Appendix F: Sample Labelling Format	31
Appendix G: DNA Extraction Protocol	32
Appendix H: Guidelines for trouble shooting DNA Contamination	43
Appendix I: Format for shipper certificate	45

List of Abbreviations:

AIIMS - All India Institute of Medical Sciences

ALL - Acute Lymphoblastic Leukaemia

Asp - Asparaginase

BM - Bone Marrow

DNA - Deoxy ribonucleic acid

EDTA - ethylene diamine tetra acetic acid

JIPMER - Jawaharlal Institute of Post-graduate Medical Education and Research

MPGx-INDALL -Molecular and Pharmacogenetic marker evaluation in relation to the toxicity and clinical response of acute lymphoblastic leukemia treatment in Indian children

MTX - Methotrexate

6-MP - 6-mercaptopurine

PK - Pharmacokinetics

RBCs - Red Blood Cells

QoL - Quality of life

TDM -Therapeutic drug monitoring

UNIGE - University of Geneva

VCR -Vincristine

CONTACT INFORMATION

The key contacts for the Project are listed below:

Dr. Uppugunduri S Chakradhara Rao,
Assistant Professor
Department of Medical oncology
JIPMER, Puducherry, India
E-mail: uscrao@jipmer.ac.in

Dr. Biswajit Dubashi
Professor, Department of Medical Oncology
JIPMER, Puducherry, India
E-mail : drbiswajitdm@gmail.com

Dr. Sameer Bakhshi
Professor
Department of Medical Oncology
AIIMS, New Delhi, India
E-mail : sambakh@hotmail.com

Dr. Marc Ansari
Associate professor
Onco-Hematology Unit
Department of Pediatrics, Gynecology and obstetrics
University of Geneva. Geneva
Switzerland
E-mail : Marc.Ansari@hug.ch

Introduction

The current study is designed to identify such genetic markers which will help in presumptive identification of future adverse events. This study is aimed to identify genetic factors which may be associated with the chemotherapy adverse effects and treatment outcome in terms of relapse and survival. We will collect germline DNA samples (from buccal swabs) and somatic DNA samples (from bone marrow) for pharmacogenetic and genetic testing. In addition, plasma and serum samples will be collected for therapeutic drug monitoring (TDM) and pharmacokinetic (PK) analysis. These samples will be obtained from two sites in India as part of the MPGx-INDALL study, which is planned to run for five years until 2025, with a possible no-cost extension until 2026.

Total number of study subjects: 500

• Number of subjects from **AIIMS**: up to 300 subjects

• Number of subjects from **JIPMER**: up to 200 subjects

Total Number of clinical samples per subject

- Buccal Swab sample for DNA: Two (***at diagnosis and on complete remission***)
- Bone marrow for somatic DNA: One+ One* (***one additional sample at the time of relapse only for the relapse cases***) # If bone marrow sample is inadequate and Pb blasts are more than 80%, Pb sample can be taken
- Whole blood for drug levels of 6MP: *One*
- Whole blood for plasma of MTX**:** ***High dose interim maintenance (only during High risk)****- One*

- Whole blood for plasma/ RBC at low dose maintenance: ***One sample of maintenance combined with that of 6 MP sample***
- Whole blood for Plasma for vincristine: One
- Whole blood for Plasma/serum for asparaginase: One
- Whole blood for Plasma Biobanking: Two (at diagnosis and on complete remission)
- Whole blood for Germline DNA- One (on complete remission)

Section-1: Preparation for sample collection:

Identification of primary sample:

Samples collected for clinical research must adhere to ICH-GCP guidelines.
https://database.ich.org/sites/default/files/E6_R2_Addendum.pdf

Reference:

WHO guidelines on drawing blood: best practices in phlebotomy especially in children: available at:
https://www.euro.who.int/__data/assets/pdf_file/0005/268790/WHO-guidelines-on-drawing-blood-best-practices-in-phlebotomy-Eng.pdf

Guidelines for Good Clinical Laboratory Practices (GCLP) - ICMR: Available at:
https://main.icmr.nic.in/sites/default/files/guidelines/GCLP_Guidelines_2020_Final.pdf

Useful information on pre-analytics:

https://mft.nhs.uk/app/uploads/2019/02/Tips-and-Tricks-in-Preanalytics-booklet.pdf

Primary sample collection:

The procedure for collection of primary samples will be described in detail within this manual.

Type and amount of the primary sample collected:

Type and amount of primary sample collected will be in accordance with the routine treatment monitoring and the study protocol.

Timings for sample collection:

Time of collection of samples is routinely noted on the proforma by the personnel collecting the sample.

Samples requiring special timing for collections are indicated within the Sample collection manual as per the requirements of the study protocol. See ***Appendix C.***

Labeling of primary samples while collecting

Samples analyzed at central laboratory are stored in cryovials. The Patient ID and initials, Type of sample (***DNA or RBC or Plasma)*** will have to be filled on the cryovials.

Site No-<br>Subject ID-<br>Int-<br>DOC-<br>DOT-<br>Sample Type-

Template of a Sample Label

Proper Identification: Label the tubes with Patient ID & Patient Initials and ensure it matches with that filled on the requisition form.

Label on sample storage tubes

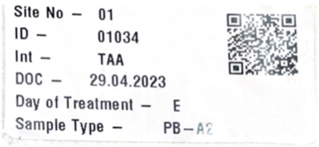

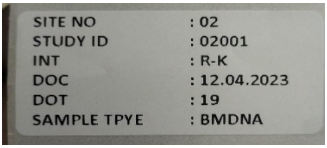

Site No- 01 (AIIMS), 02 (JIPMER)

ID = Site No Patient No (eg., First patient of JIPMER- 02001 and First patient of AIIMS -01001)

Int: Initials of First Name Second Name Last Name

For Example: XYZ, X_Z (if second name not available)

DOC- Date of Collection (DD.MM.YYYY)

DOT-Day of Treatment (For e.g. I9 (For day nine of Induction))

(*Enrollment: E; Induction: I; Maintenance: M; Consolidation: C1 (MRD negative at the end of induction); End of Consolidation: EC (MRD negative at the end of consolidation); Interim Maintenance: IM*)

Sample Type:

**Sample Type**	**Code**
*Buccal Swab Germline DNA	BSGDNA
*Bone Marrow Somatic DNA	BMSDNA
*Whole Blood Somatic DNA	WBSDNA

*Whole Blood Germline DNA	WBGDNA
Enrolment Plasma Bio banking	PB-A1,2,3
Plasma Vincristine	Plasma VCR
Plasma/Serum L-Asparaginase	Plasma/Serum L-asp
Remission plasma Bio banking	PB-B1,2,3
Plasma High Dose Methotrexate	PBHDMTX
RBC for Low Dose Methotrexate and 6-MP	RLDMTX /R6MP
DNA Aliquots	Suffix 1,2,3 for eg. BMSDNA1
QC Check (Qubit)	Suffix QC for eg. BMSDNAQC
DNA concentration (Nanodrop)	Suffix ND for eg. BMSDNAND

** Include the quantity of the **DNA in ng/μL** in addition to the volume in parenthesis ().*
Refer to Appendix A1 for Flowchart describing details of Specimen required, time points and tubes for collection, and storage
Refer to Appendix F for Sample Labelling format

Section-2: Collection of blood samples

A. Routine Sampling

1. *Please note that some of the sampling is a part of routine clinical management and not to be considered as part of the study sampling, but the data collected will be used for the study Appendix E.*
2. *Overall schedule of assessments is listed in Appendix B.*

B. Study Specific Sampling

1. General instructions

- Each type of sample collected i.e., plasma, serum, RBC and DNA must be stored in separate cryoboxes labelled with the study name and with sample type on all sides of the cryoboxes.
- The consumables required for each type of sample collection are listed in *Appendix A2* and a summary of aliquots and storage conditions for each time point is given in *Appendix D*.
- All details regarding the collected sample and its storage must be entered in a master sheet created for this purpose. This includes Subject name, UHID/Hospital ID, Day and date of collection, and storage location details (Freezer location, shelf, box number and name).

2. Drug level measurement

Plasma separation for biobanking, VCR, HDMTX, LDMTX

Procedure:

- Collect 3.0 ml of blood in BD EDTA K2 vacutainer using standard techniques (vacutainers with 4ml or 6ml collection capacity).
- Invert tube gently 3-4 times to mix sample with EDTA K2.
- Place tube on crushed ice immediately
- Centrifuge at 1000×g (rcf) for 15 min (cold)
- Separate plasma without disturbing buffy coat and make 3 aliquots of 400uL each (For remission sample, take out whole plasma leaving 100ul in the vial. Store the vial as it as for germline DNA extraction)
- Store the samples in an Ultra-Low Temperature freezer at -80^{0}C.

Note: *Use centrifuges with swing buckets always and at 4^{0}C for centrifugation and separation of plasma.*

Sample collection timepoint:

- Collect blood samples 24 hours after the end of infusion to measure vincristine drug levels during the induction phase.
- To measure L-Asparaginase drug levels during the induction phase, collect blood samples 72 hours after infusion for Native E. coli L-Asparaginase and 7 days after infusion for Peg L-Asparaginase.
- During Interim Maintenance therapy (for high-risk patients only), collect whole-blood samples 48 hours after the end of high-dose methotrexate infusion and separate the plasma.

RBC for 6MP, LDMTX

Procedure:

- After centrifugation of blood sample in EDTA vacutainer (described above) at 1000×g (rcf) for 15 minutes in refrigerated swing bucket centrifuge, remove the top layer and aliquots as instructed. Carefully remove plasma and buffy coat till RBC layer is reached.
- Now pipette bottom RBC layer (600-800 µl) into 15 ml Falcon tube
- Check RBC count in aliquot* Add 5X volume of HBSS to RBC pellet and Centrifuge at 1000×g (rcf) for 60-90 seconds
- Repeat wash
- Pipette out the supernatant and discard#
- Resuspend RBC in HBSS to ~8×10^8 cells/200 µl (Erythrocyte count determined before freezing will be used to normalize metabolite concentrations to pmol/8×10^8 RBCs)
- Divide RBC in two/three aliquots of 200 µL each.
- Transfer RBCs into labelled cryovials and store at -80°C in ULT freezer.

Sample collection timepoint:

- Collect whole-blood samples during the eighth week of maintenance therapy (i.e., between days 49 and 56) to measure 6-mercaptopurine, 6-thioguanine, and 6-methyl mercaptopurine levels in the whole blood matrix or RBCs.

**To be done at oncology lab in an automated counter by lab technician*
# Discard wash supernatant without disturbing RBC pellet and mention RBC count on the cryovials

Serum for LASP

Procedure:

- Collect 3.0 ml blood in BD SST vacutainer.
- Gently invert the tube 6-8 times immediately after collection. The blood sample collected should be allowed to clot for 15-30 minutes at Room temperature.
- Centrifuge the tube at 1000×g (rcf) for 15 minutes.
- Using the pipette, transfer serum equally into three Cryovials of volume 400 µl each.
- Immediately freeze & store the samples at -80°C.

Sample collection timepoint:

- Collect blood samples on day 3 after the first infusion during early induction using SSL tubes for serum.

Note: *Do not delay holding time for clot separation as this increases chances of hemolysis.*

Important note: Due to stability issues all the samples for drug level measurements must be shipped in the recommended temperature conditions to the analytical laboratory within 3 months

form the date of collection for analyses. Minimum number of samples required for shipment if it is before 2 months = 20.

DNA from Buccal Swab/Saliva kit

Saliva/buccal swab sampling instructions

Procedure:

- Collect buccal swab sample from a patient until at least 30 minutes after they have drunk, eat, or chewed gum. Ensure donor is in an upright position during sample collection. If this is not possible, please make a note on the Form accompanying the sample. If possible, avoid rubbing the teeth. Follow the instructions mentioned below.
- Buccal Swabs can be used for patients under 2 years old and collection kits can be used for more than 2 years old, but for patients failing to give saliva sample, swabs can be used as an alternate way for collection

Note: *If the patient is suffering from mucositis, has gum bleeding, mouth ulcers or is on chemotherapy, postpone collection until these symptoms have resolved and the course of chemotherapy is completed.*

Pediatrics-OC-175- (for children below 2 years)

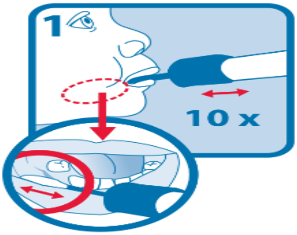

1. Open package and remove collector without touching sponge tip. Place sponge as far back in the mouth as comfortable and rub along the lower gums (see close up image) in a back-and-forth motion. Gently rub the gums 10 times. If possible, avoid rubbing the teeth.

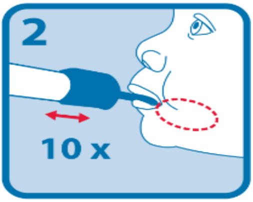

2. Gently repeat rubbing motion on the opposite side of the mouth along the lower gums for an additional 10 times.

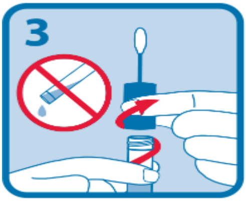

3. Hold the tube upright to prevent the liquid inside the tube from spilling. Unscrew the blue cap from the collection tube without touching the sponge.

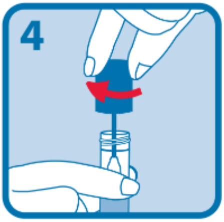

4. Turn the cap upside down, insert the sponge into the tube and close cap tightly.

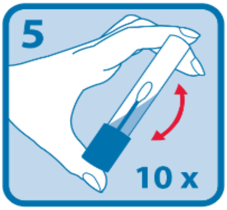

5. Invert the capped tube and shake vigorously 10 times.

Adult- OG-575- (for children above 2 years)

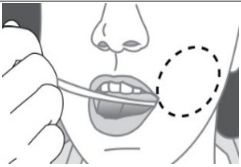

1. Place one sponge in cheek pouch. Gently move the sponge along the gums and inner cheeks for 30 seconds to soak up as much saliva as possible.

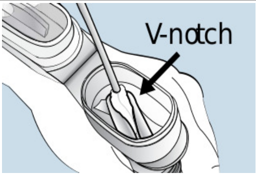

2. Once sponge is saturated with saliva, insert sponge in V-notch of funnel. Wring saliva out of sponge using a twisting and pushing motion against the inner wall of the V-notch. Saliva will flow into tube.

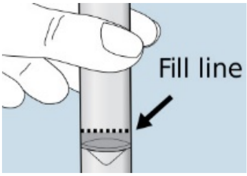

3. Repeat these steps (1 to 2) USING THE SAME SPONGE until the liquid saliva (not bubbles) reaches the fill line. Check sponge for damage each time before inserting into donor's mouth. Use second sponge if first sponge shows any signs of wear or tear. Tap tube bottom against hard surface to reduce number of bubbles.

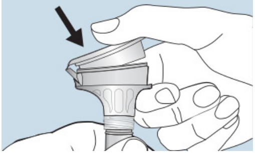

4. Hold the tube upright with one hand. Close the lid with the other hand (as shown) by firmly pushing the lid until you hear a loud click. The liquid in the lid will be released into the tube to mix with the saliva. Make sure the lid is closed tightly.

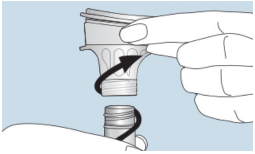

5. Hold the tube upright. Unscrew the funnel from the tube.

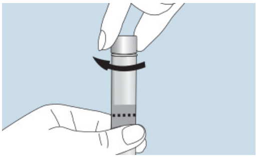

6. Use the small cap to close the tube tightly.

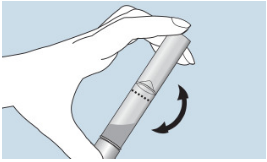

7. Shake the capped tube for 5 seconds. Discard or recycle the funnel. Discard sponges.

Sample collection timepoint:

- Collect sample at the time of enrollment and at the time of remission

Sample storage:

Saliva sample shall be stored at room temperature in a box or at 4^0C (regular refrigerator) until shipment or extraction of DNA

If DNA has been extracted from MRD laboratory, store and send them frozen (Label information must be respected as given in page 9).

To order more sampling kits and packaging supplies please contact CANSEARCH Research platform for pediatric Oncology and Hematology at University of Geneva.

DNA: BMDNA (From Bone Marrow sample) *

Procedure:

- Put 500µl aspirated bone marrow sample in purple top microtainer
- Invert and mix gently 3-4 times and transfer to 15 ml falcon tube and add 3 times the volume of cold RBC LYSIS buffer to it and shake vigorously for 4-5 seconds.
- Let it stand for 45-50 minutes at Room Temperature.
- Centrifuge# at 1000×g (rcf) for 10 minutes at room temperature and discard the supernatant using a P1000 pipette
- Repeat RBC LYSIS buffer step for 15 minutes after gentle disruption of pellet and centrifuge at 1000×g (rcf)
- Remove supernatant# and add 1ml 1X PBS buffer and centrifuge at 1000×g (rcf) for 10 minutes at room temperature
- Remove supernatant,
- Check pellet volume (use small p200 tip with P200), divide into two ALIQUOTS and transfer pellet to two labelled cryovials
- Store in ULT freezer at -80°C.

# Take care not to disturb the pellet
** This step will be done at Department of Biochemistry, AIIMS, New Delhi.*
** This step will be done at the Medical Oncology laboratory, JIPMER, Puducherry*

Sample Collection Timepoint :

- Baseline (at recruitment) and at relapse/MRD

- Bone Marrow for DNA during start of Induction is mandatory.
- Bone marrow samples will be collected in EDTA K2 microtainer tubes (Lavender Cap)
- They are to be processed for RBC lysis followed by washing and storage of WBCs as detailed below.
- Aliquots shall be kept frozen at -80°C until extraction.

Section-3: DNA Extraction & Quality check

Kindly refer to *Appendix- G for DNA extraction protocol and for troubleshooting refer to Appendix- H*

Quality control of genomic DNA:

1. Amount required for the library preparation and sequencing:

GENERAL: Quantity measured by qubit having minimum of 300 ng of input material is recommended at a minimum concentration of 2ng/uL. Though sometime with 150 ng input, also we can perform but not preferred.

2. Checking the quality and contamination of proteins or RNA:

Measure the Absorbance at 260, 280, and 230 nm and then take the ratios of 260/280 (between 1.8-2) and 260/230 (should be greater than 2). In our laboratory, we will use more sensitive fluorometric method using Qubit fluorometric quantitation (Invitrogen), which has specific kit for double strand DNA and can be used with specific standards (reliable method). However, to rule out RNA contamination nanodrop also will be used to measure 260 and 280 absorbance (only 2μL of sample will be used for this purpose).

3. Checking for integrity:

The integrity of gDNA samples is usually assessed by running 2-5µL of each sample on 0.8-1% agarose gel(s) against a 1kb DNA ladder or in our case we will run them on tape station which required very low volumes (1-2µl). gDNA should have a high molecular weight band (>12kb) with little/no evidence of degradation (smearing) and minimal RNA contamination.

Note: *The library preparation with TrueSeq DNA PCR free preparation kit, which recommended if we have the quantity of DNA between 1- 2µg. However, it seems if we must obtain samples stored or from some material collected previously (with low quantities), then we must specify in the proposal to use an alternate library preparation (True Seq nano for e.g.)*

Library preparation could be uniform to avoid methodology variations. PCR free library is recommended for de novo sequencing and is mostly free of any errors. But if we include the verification of variants by another procedure after sequencing for e.g. real time PCR (additional step), then we can choose a library preparation kit which require very low concentrations of DNA (in ng).

4. Requirement for Whole exome / genome sequencing (mainly for whole genome):

Here are the minimum gDNA requirements. Concentration of 1.5-2.0 µg gDNA will be preferred.

- 1µg of gDNA in 50µl 10mM Tris (8.0). If the Genome Centre must perform the DQN/DIN analysis, please provide 1.1µg in 55ul.
- DNA concentration MUST be determined by Qubit
- DIN/DQN value greater than 7.5
- Please provide 260/280 and 260/230 ratio

Section- 4: Courier of samples for drug level measurement

Shipment instructions for Drug level Measurement.

- Samples should be sent at a minimum every 3 months.

Contact details of the Drug level measurement laboratory:
Prof. Jayanthi
Department of Pharmacology
Jawaharlal Institute of Post-Graduate Medical Education and Research (JIPMER)
Puducherry- 605006
E-mail: drjayanthi2008@gmail.com
Tel: +919442395291

For L-asp and methotrexate drug levels measurement (at AIIMS)
Dr. Archna Singh
Department of biochemistry,
Room Nos. 3044 and 3046a,
AIIMS, New Delhi-110029, India
Tel: +91-11-26593635; 26593478
E-mail: arch_singh@ymail.com, arch2574@gmail.com

Section- 05: Sample Preparation and Packaging

- Kindly Verify labels accurately before packaging.
- Prepare a data sheet.
- Sample should be stored in sample holder box (thermocol or directly place the sample in plastic bag that is appropriately insulated for damage.
- Place dry ice or gel ice to cover the samples in their entirety (above and below) and make sure that they do not move while shipment causing friction on the samples and tightly placed.
- Pack it in thermocol box and then in a carton box and duly place the documents required along with the destination address and sender's address.
- Inform the laboratory at least one week in advance (if it is 2 weeks much better) before shipment, to plan and schedule the work

Checklist for Sample packaging:

- Verify sample type (e.g., blood, plasma, serum, buccal, bone marrow)
- Confirm sample labelling with patient ID, date, and sample type
- Check sample volume and condition (no haemolysis, contamination, or leakage)
- Prepare necessary documentation (sample collection form, requisition forms, shipper certificate enclosed as *Appendix I*)
- Confirm samples are within the required temperature range for transport

- Check package integrity (no damages, leaks)
- Verify compliance with all transport regulations
- Arrange timely shipment to the testing laboratory

Section - 6: Instructions on courier of DNA samples

After extraction, aliquot DNA into tubes 1µg/50µL into each tube in tris buffer for long term storage.

Phase 1 (all samples)/2 (partial samples for quality testing): Sample shipment to CANSEARCH Research Platform of Pediatric Hematology and oncology of University of Geneva

Please get in contact two weeks before shipping the samples, which will allow CANSEARCH Research Laboratory to arrange the shipment with the preferred courier for your center

Please ship samples to the following address:
Prof. Marc Ansari,
CANSEARCH Research Platform in Pediatric Oncology and Hematology,
Department of Pediatrics, Gynecology and Obstetrics, University of Geneva,
Faculty of Medicine
Centre Medical Universitaire - 1211 Genève 4
Switzerland

Appendix A1: Flowchart for Specimen Collection time points, sample type and storage

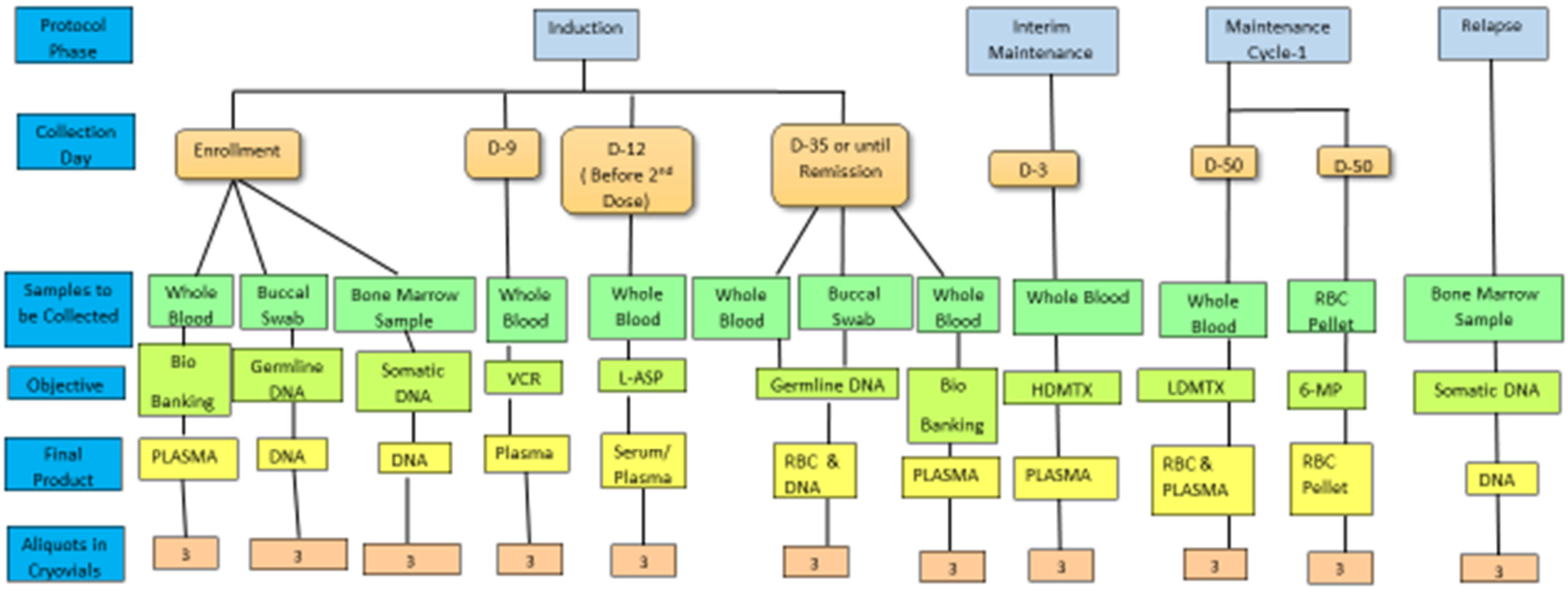

If first infusion collection time point is missed. Drug related sample can be taken on subsequent dosages with reason of first dose missed to be mentioned.

Each aliquot should contain 400ul not less that if sample volume is less, reduce the number of aliquots but not the volume.

If MRD is positive at the end of induction. Repeat bone marrow is done at the end of consolidation and if MRD is negative, remission whole blood /plasma biobanking sample is taken at the end of consolidation.

Appendix A2: List of consumables required for a) **Blood** and b) **Bone marrow** collection and processing: (Indicate catalogue numbers of your local supplier for a quick reference)

a. **BLOOD**

**Chemicals and Reagents**	**Make**	**Catalog Number**
Hanks' balanced salt solution (procure commercially)	Himedia	TL1109-100ML
Vacutainers 4 or 6 ml	BD	367844, 367861
Spare vacutainers for balance in centrifuge	BD	367844, 367861
Microchannel pipettes	Tarson	30010 (1-10ul), 30020 (2-20ul), 30040 (20-200ul), 30050 (100-1000ul)
Micro tips	Tarson	(521000-10ul, 521010-200ul, 521020-1000ul)
Cooling centrifuge	Eppendorf	
2 mL microcentrifuge tubes	Tarson	500020
Cryovials	Tarson	523051
Cryolabels	TSC	TE244
Cryomarkers	Himedia	LA697A-1NO
Cryoboxes for storage	Tarson	202070
Discard pot/beaker for buffers/tips, etc.,	-	-

b. **BONE MARROW**

**Chemicals and Reagents**	**Make**	Catalog Number
RBC Lysis buffer	Himedia	R075-100ML
Vacutainers 4 or 6 ml	BD	367844, 367861
Microchannel pipettes	Tarson	30010 (1-10ul), 30020 (2-20ul), 30040 (20-200ul), 30050 (100-1000ul)
Micro tips	Tarson	(521000-10ul, 521010-200ul, 521020-1000ul)
Stands for placing microtainer	Tarson	
15 ml falcons for RBC lysis and balancing during centrifugation	Tarson	500031
Cooling centrifuge	Eppendorf	
Cryovials	Tarson	523051
Cryolabels	TSC	TE244
Cryomarkers	Himedia	LA697A-1NO
Cryoboxes for storage	Tarson	202070

Appendix B: Schedule of Assessments

Procedures	Information	Enrollment	Treatment using IciCLE protocol with risk stratification into Standard risk (SR, Intermediate risk (IR), and high risk (HR). treatment duration and time lines mentioned in terms of weeks (W)						Retrospective data collection post treatment (1 year+)	
		Day 0	Induction (1-5 weeks)	Consolidation (6-8W -SR; 6-10W-IR; 6-14W-HR)	Interim-maintenance 9-17W-SR;11-18W-IR;15-22W-HR	Delayed intensification 18-24-SR; 19-25W-IR;23-29W-HR	Maintenance 1st cycle of 12W (25W-36W-SR);26 W-37W-IR; 30W-41W-HR	Maintenance 1st W of the 2nd cycle (37W-SR; 38W-IR; 42W)	11 weeks of 2nd cycle + 6 cycles of 12 weeks each.	Follow-up visits until one-year post treatment
Eligibility Screen	X									
Risk stratification	X									
Informed consent	X									
Biological sampling		X		X					X	X
Nutritional assessment		X	X	X	X	X	X	X	X	X
Socio-Economic status assessment		X								
Participant characteristics & History		X								
Toxicity data collection*			X	X	X	X	X	X		
Complete blood profile, LFT, RFT**			X	X	X	X	X	X	X	
Steroid response			X							
Drug level Measurement			X		X		X			

Relapse and Survival outcomes data			X	X	X	X	X	X	X	X
Quality of Life PedSQl assessment		D0-D15 →	X	X	X			X		
SAE collection and reporting		X	X	X	X	X	X	X		
Standard clinical care as per IciCLE protocol		X	X	X	X	X	X	X	X	

* Toxicity data collection using the Common Terminology Criteria for Adverse Events available at: (CTCAE-version 5.0 – https://ctep.cancer.gov/protocolDevelopment/electronic_applications/ctc.htm#ctc_50)

** part of routine clinical care, assessed one week during induction until maintenance and then once in every two weeks during maintenance phase for the first 100 days. After the first week of second cycle it will be assessed on each patient follow-up visit, not included in the study data collection.

W-Week; SR-Standard risk; IR-intermediate risk; HR-High risk;

Appendix C- Study specific sampling details: Real time shipment to courier (internal within the institute or to an analytical laboratory or Pathology laboratory) - Keep one sheet in patient source files with the ID of the patient (To be put with sample log form in paper CRF)

Phase	Day of sampling	Samples to be collected	Purpose	Sample	Collection Tube and Volume	Transferred to cryovials	$Volume of aliquot (No of aliquots)	Storage After processing	Check Y/N	Remarks
Enrollment /Screening	0	*Bone Marrow	Somatic DNA Analysis/ Biobanking	Somatic DNA	EDTA K2 microtainer- 500 uL	Yes	NA	-80^{0}C (ULT)		
	0	Peripheral blood Sample	Plasma Biobanking	Plasma	EDTA K2 vial 3.0 ml	Yes	400ul	-80^{0}C (ULT)		
	0	Buccal Swab	Germline DNA Analysis/ Biobanking	Germline DNA	1 (OC-175 / OG-575)	No	NA	4^{0}C (ULT)		
Induction	9*	Whole Blood	Drug levels measurement	Plasma	EDTA K2vial 3.0 ml	Yes	400ul	-80^{0}C (ULT)		
	72 hours after First Dose	Whole Blood	Drug levels measurement	Serum	SST vial 3.0 ml	Yes	400ul	-80^{0}C (ULT)		
	35 or at the time of remission	Buccal Swab	Germline DNA Analysis/ Biobanking	Germline DNA	1 (OC-175 / OG-575)	No	NA	4^{0}C (ULT)		
		Peripheral blood Sample	Plasma Biobanking and Whole blood	Plasma and Whole blood	EDTA K2 vial 3.0 ml	Yes	400ul	-80^{0}C (ULT)		
Interim Maintenance	3#	Whole Blood	Drug levels measurement	Plasma	EDTA K2 3.0 ml	Yes	400ul	-80^{0}C (ULT)		

Maintenance	Between 50-56	RBC count must be tested	Drug levels measurement	RBC Pellet	EDTA K2 3.0 ml	Yes	200ul	-80^{0}C (ULT)		
			TDM	RBC and Plasma	EDTA K2 3.0 ml	Yes	400ul	-80^{0}C (ULT)		
At the time of Relapse		Bone Marrow	Somatic DNA Analysis/ Biobanking	Somatic DNA	EDTA K2 microtainer- 500ul	Yes	400ul	-80^{0}C (ULT)		

*This step will be done at Dr. Jayanth's lab at Biochemistry, AIIMS, New Delhi.

@ if delayed then 72hrs after the first infusion
* if delayed then 24hrs after the first infusion,
# if delayed then 48hrs after the first infusion

- After extraction Quantity of DNA aliquot should be 1µg/50µL

Appendix D: Summary of storage, processing and transport logistics. All samples are to be stored in cryovials (500 mL)- keep this information in source file with patient unique ID and update eCRF

OBJECTIVE	Sample Type	Sample Volume	Final Product	Final product volume	Final Product concentration	Aliquots (ul)	Storage	Transport	Kits	Total Nos of Cryovials	Total no of Vacutainers
**Somatic DNA**	*Bone Marrow	500uL	DNA			2 (5ul)<br>1(1ug)	-	√	QiAmp DNA Blood Kit	1 + 2 (1= Primary storage 2 = Aliquots)	1
			DNA (In case of Relapse)			2 (5ul)<br>1(1ug)			QiAmp DNA Blood Kit	1 +2	1
**Germline DNA**	Buccal Swab	-	DNA			2 (5ul)<br>1(1ug)	-	√	QiAmp DNA Blood Mini Kit	1+2	1

Appendix E: Routine laboratory testing- keep this information in patient source file

Phase	Risk Stratification (Standard risk- SR, Intermediate risk- IR, High Risk- HR)	**Week (numbering from the beginning of the phase)**							Tests Performed	Type of Tube	Remarks
Induction	Same for all	☐ 1	☐ 2	☐ [illegible]	☐ 4	☐ 5			CBC	EDTA	
Consolidation	SR	☐ 1		☐ 3							
	IR	☐ 1		☐ 3							
	HR	☐ 1	☐ 3	☐ [illegible]	☐ 7						
Interim Maintenance	SR	☐ 1	☐ 3	☐ [illegible]	☐ 7	☐ 9			LFT, KFT, Amylase, Lipase, FBS	SERUM	
	IR	☐ 1	☐ 3	☐ [illegible]	☐ 7						
	HR	☐ 1	☐ 3	☐ 5							
Delayed Intensification	Same for all	☐ 1	☐ 3	☐ 5							
Maintenance	Same for all	☐ 1	☐ 3	☐ [illegible]	☐ 7	☐ 9	☐ 11	☐ 13	CBC LFT, KFT	EDTA SERUM	

Appendix F: Sample labelling format

DAY	Phase	Day of treatment	Sample to collected	Processing sample	Sample ID
Day 0	Enrolment	E0	Bone Marrow	Bone Marrow	BMSDNA
			Whole Blood	Plasma Biobanking	PB-A (1,2,3)
			Buccal swab	Buccal Swab	BSGDNA
Day 9	Induction	I9	Whole Blood	Plasma	Plasma (VCR)
Day 12	Induction	I12/I16	Whole Blood	Serum/Plasma	Serum/plasma (L-ASP)
Remission (MRD negative)	End of induction/ end of consolidation	C1/ EC	Whole Blood	Plasma Biobanking	PB-B (1,2,3)
				DNA	WBSDNA
			Buccal Swab	Buccal Swab	BSGDNA
Day 3	Interim Maintenance	IM3	Whole Blood	Plasma	PBHDMTX
Day 50	Maintenance	M50	Whole Blood	Plasma	PBLDMTX
				RBC	RLDMTX
			Whole Blood	RBC	R6MP

Appendix G: DNA Extraction Protocol

REAGENT GRADE

1. 100% Absolute ethanol, Grade- ACS, ISO, Reag. Ph Eur, (EMSURE Make, CAS #-64-17-5)
2. PBS Tablets- Each PBS tablet should be dissolved in 100 mL of distilled water. This buffer contains 10 mM phosphate, 150 mM sodium chloride, pH 7.3 to 7.5. (Invitrogen Make-Cat. No.- 003002)
3. QIAamp Blood Mini Kit (**Cat. No-51304)** for all three protocols.

BASIC PRECAUTIONS/GENERAL INSTRUCTIONS

1. When working with chemicals and patient samples, always wear a suitable lab coat, disposable gloves, and protective goggles.
2. Double sequential Elution should be done properly from the same column so as to get maximum DNA yield.
3. All plastic wares (tips, MCT) should be sterile before use to avoid contamination.
4. Mix Buffer AL thoroughly by shaking before use.
5. Do not add QIAGEN Protease or proteinase K directly to Buffer AL.
6. Before using AW1 and AW2 buffers for the first time, add the appropriate amount of ethanol (96-100%) as indicated on the bottle*.
7. Small samples should be adjusted to 200 µL with PBS before loading.
8. Avoid overloading the column otherwise it will clog/saturate the silica membrane.
9. Avoid touching the QIAamp membrane with the pipet tip as this can damage it.
10. After all pulse-vortex steps, briefly centrifuge the 1.5 mL micro centrifuge tubes to remove drops from the inside of the lid.
11. Change pipet tips between all liquid transfers to avoid cross contamination.
12. Pipet the sample into the QIAamp Mini column without wetting the rim of the column.

13. LABELLING: Aliquots from all types of samples stored for concentration and QC check (NanoDrop, Bioanalyzer, and Tapestation) should be labelled as per the labeling instructions mentioned in the lab manual with a suffix of ND for nanodrop and QC for the QC check samples. *Hence, Sample should be labelled with aliquot number or QC sample (Suffix as 1/2/3/ for aliquot and ND/QC after sample type, e.g., BMDNA1; BMDNA-ND/QC.*

* ***if you are not going to use all of columns and hence the buffer in a single run, please take only the required amounts of buffer in a separate, clean container and dilute with appropriate amounts of ethanol; else the buffer composition will change as ethanol will evaporate by the time of your next run.***

Whole Blood DNA Extraction Protocol (QIAamp-Kit based Cat. No-51304)

(Note: Use 200ul of sample for single spin column, and use different column for remaining leftover samples)

Add 20µl Proteinase K in 200µl MCT

↓

Add 200µl whole blood and 200µl PBS to the MCT

↓

Add 200 µl AL Buffer to the above

↓ Pulse vortex for **15 seconds**

Incubate at 56°C for 25 minutes (dry bath/water bath using a floater)

↓ Briefly Centrifuge for spin down (**4000 rpm for 3-4 secs**)

Add 200µl ethanol (96-100%)

↓ Pulse vortex for **15 seconds**

Transfer the whole volume to a mini spin column (placed in a 2ml collection tube)

Centrifuge tube with column at **8000rpm (RT)** for **1 minute**

↓

Discard the collection tube and place the column in the new collection tube

↓

Add 500µl AW1 buffer to the column

↓ Centrifuge at **8000rpm (RT)** for **1 minute**

Discard the collection tube and place the column in the new collection tube

↓ Add 500µl AW2 wash buffer

Centrifuge at **14000rpm** (RT) for **3 minutes**

↓

Repeat Centrifuge at **14000rpm** for **3 minutes** without adding wash buffer.

↓

Discard the collection tube and place the column in the new collection tube.

Place the mini-column in the **new 1.5ml MCT**, add 100µl AE buffer

↓

Incubate at **(RT)** for **20 minutes** and centrifuge at **8000 rpm** for **1 minute**

↓

Add 50µl AE buffer in the same column and incubate at **(RT)** for **20 minutes** and repeat centrifuge at **8000 rpm** for **1 minute**

↓

Collect the eluted DNA in 1.5 ml MCT

Take 2µ1 DNA sample for checking quality and DNA concentration

Check using Nano Drop (both at 260/280 nm and 260/230nm)

<u>PLEASE USE THE P2.5 MICROCHANNEL PIPETTE FOR THIS WORK</u>

Make 3 aliquots (of total 1µg DBA each) for storage -80°C and transfer to Geneva for sequencing

E.g. Elution volume 150µl, minimum conc. 25 ng/µl (1µg=1000 ng, 1000/25=40) Then, make 3 aliquots of - 120µl DNA (each 40µl), and one 10µl DNA aliquot for quality and conc. (NanoDrop. Bioanalyzer and Tapestation).

Sample should be labelled as aliquot or QC sample (Suffix as 1/2/3/ for aliquot and ND/QC after sample type. E.g. BMDNA1; BMDNA-ND/QC)

Bone Marrow DNA Extraction Protocol (QIAampKit based Cat. No-51304)

(Note: Use 200ul of sample for single spin column, and use different column for remaining leftover samples)

The maximum number of cells to use is 5 x10^6

(*Cells count approximation by bone marrow blast percentage and sample volume taken*)

↓

Add 20µl Proteinase K in 200µl MCT

↓

Add WBC cell pellet and 200µl PBS

↓

Add 200µl AL Buffer

↓ Pulse vortex for **15 seconds**

Incubate at **56°C for 25 minutes**

↓ Briefly Centrifuge

Add 200µl ethanol (96-100%)

↓ Pulse vortex for **15 seconds**

Transfer the whole volume in a mini spin column (2ml collection tube)

↓ Centrifuge at **8000rpm** for **1 minute**

Add 500µl AW1 buffer

↓ Centrifuge at **8000rpm** for **1 minute**

Add 500µl AW2 wash buffer

↓ Centrifuge at **14000rpm** for **3 minutes**

Repeat Centrifuge at **14000rpm** for **3 minutes** without adding wash buffer

↓

Place the mini-column in the new 1.5ml MCT, add 100µl AE buffer,

↓ Incubate at **(RT)** for **20 minutes** and centrifuge at **8000rpm** for **1 minute.**

Add 50µl AE buffer in the same column,

Incubate at **(RT)** for **20 minutes** and repeat centrifuge at **8000rpm** for **1 minute.**

↓

Collect the DNA sample in 1.5 ml MCT

Take 2µ1 DNA sample for checking quality and DNA concentration

Check using Nano Drop (both at 260/280 nm and 260/230nm)

PLEASE USE THE P2.5 MICROCHANNEL PIPETTE

Make 3 aliquots (of total 1µg DBA each) for storage -80°C and transfer to Geneva for sequencing

E.g. Elution volume 150µl, minimum conc. 25 ng/µl (1µg=1000 ng, 1000/25=40) Then, make 3 aliquots of - 120µl DNA (each 40µl), and one 10µl DNA aliquot for quality and conc. (NanoDrop. Bioanalyzer and Tapestation).

Sample should be labelled as aliquot or QC sample (Suffix as 1/2/3/ for aliquot and ND/QC after sample type. E.g. BMDNA1; BMDNA-ND/QC)

Buccal Swab DNA Extraction Protocol (QIAampKit-based Cat. No-51304)

(Note: Use 200ul of sample for single spin column, and use different column for remaining leftover samples)

Add 20µl Proteinase K in 200µl MCT

↓

Add 400µl Buccal swab sample (same volume from both types of collection kits)

↓

Add 200µl AL Buffer

↓ Pulse vortex for **15 seconds**

Incubate at **56°C** for **25 minutes**

↓ Briefly Centrifuge

Add 600µl ethanol (96-100%)

↓ Pulse vortex for **15 seconds**

Transfer the whole volume in a mini spin column (2ml collection tube)

↓ Centrifuge at **8000rpm** for **1 minute**

Add 500µl AW1 buffer

↓ Centrifuge at **8000rpm** for **1 minute**

Add 500µl AW2 wash buffer

↓ Centrifuge at **14000rpm** for **3 minutes**

Repeat Centrifuge at **14000rpm** for **3 minutes** without adding wash buffer

↓

Place the mini-column in the new 1.5ml MCT, add 100µl AE buffer

Incubate at **(RT)** for **20 minutes** and centrifuge at **8000rpm** for **1 minute**

↓

Add 50µl AE buffer in the same column

↓ Incubate at **(RT)** for **20 minutes** and repeat centrifuge **8000rpm** for **1 minute**

Collect the DNA sample in 1.5 ml MCT, quality check by NanoDrop (260/280 and 260/230), and storage of the sample at **-80°C**.

↓

Collect the DNA sample in 1.5 ml MCT

Take 2µ1 DNA sample for checking quality and DNA concentration

Check using Nano Drop (both at 260/280 nm and 260/230nm)

<u>PLEASE USE THE P2.5 MICROCHANNEL PIPETTE FOR THIS WORK</u>

Make 3 aliquots (of total 1µg DBA each) for storage -80°C and transfer to Geneva for sequencing

E.g. Elution volume 150µl, minimum conc. 25 ng/µl (1µg=1000 ng, 1000/25=40) Then, make 3 aliquots of - 120µl DNA (each 40µl), and one 10µl DNA aliquot for quality and conc. (NanoDrop. Bioanalyzer and Tapestation).

Sample should be labelled as aliquot or QC sample (Suffix as 1/2/3/ for aliquot and ND/QC after sample type. E.g. BMDNA1; BMDNA-ND/QC)

Appendix H: Trouble shooting guidelines for Low grade DNA/Contamination

Purpose: In quality analysis of DNA, the ratio of A260/A280 is optimal at 1.8. If the A260/A280 ratio is <1.8, the contaminant is protein (follow the SOP for removing protein contamination from the DNA samples using Proteinase K). If the A260/280 ratio is >1.8, the contaminant is RNA (follow the SOP for removing RNA contamination from the DNA samples).

1. **SOP for removing Protein contamination from the DNA samples using Proteinase K.**

- Set the dry bath at 56°C.
- Take 50µL of DNA samples (Protein contaminated samples) in a vial.
- Add 5µL of Proteinase K (1mg/mL) to the sample vial.
- Incubate at 56°C for 60 minutes using dry bath.
- Add 200µL (97% to 100%) ice-cold ethanol to the mixture to precipitate the DNA.
- Load the sample to a mini spin column (placed in a 2mL collection tube).
- Centrifuge the tube with column at 8000rpm (RT) for 1 minute.
- Discard the collection tube and place the column in a new collection tube.
- Add 500µl AW1 buffer to the column.
- Centrifuge the tube with column at 8000rpm (RT) for 1 minute.
- Discard the collection tube and place the column in the new collection tube.
- Add 500µl AW2 wash buffer.
- Centrifuge at 14000rpm (RT) for 3 minutes.
- Repeat Centrifuge at 14000rpm for 3 minutes without adding any buffer.
- Discard the collection tube and place the mini-column in the new 1.5ml MCT, add 100µl AE buffer.
- Incubate at (RT) for 30 minutes and centrifuge at 8000 rpm for 1 minute

- Collect the eluted DNA and quantify to evaluate the removal of proteins.

2. SOP for removal of RNA contamination from the DNA samples using RNase-A.

- Take 50µL of DNA samples (RNA contaminated samples) in a vial.
- Add 10µL of RNase A to the sample.
- Incubate at 37°C for 60 minutes.
- Add 6µL of 3 M sodium acetate to RNase A treated DNA
- Add 200µL (97% to 100%) ice-cold ethanol to the mixture to precipitate the DNA.
- Place at -80°C for 30 minutes (or -20°C for overnight)
- Centrifuge the sample at 4°C for 20 minutes to pellet DNA
- Carefully, pour off supernatant and wash the pellet with 70°C ethanol (ice-cold)
- Centrifuge the sample at 4°C for 3-5 minutes
- Discard the supernatant and air dry the pellet.
- Resuspend the pellet with 50µL elution buffer.
- Quantify and qualify the DNA and store it at -80°C.

Appendix I: Format for Shipper Certificate

Gram:"JIPMER"
Website: www.Jipmer.edu

Phone: 2272380-90
Fax: 0413-2272067

JAWAHARLAL INSTITUTE OF POSTGRADUATE MEDICAL EDUCATION AND RESEARCH
Institute of National Importance (Government of India)
Ministry of Health and Family Welfare,
Dhanvantri Nagar, Puducherry-6

Dated:

SHIPPER'S CERTIFICATION FOR NON-HAZARDOUS CARGO

AIR/ SEA/ ROAD SHIPMENT NO	PORT OF DEPARTURE	PORT OF DESTINATION
	**JIPMER** Dhanvantri Nagar, Gorimedu, Puducherry- 605006, India.	

This is certify that the articles/substance of this shipment are properly described by name, that they are not listed in the current edition of IATA Dangerous Goods Regulations (DGR) Alphabetical list of Dangerous Goods, nor do they correspond to any of the Hazard Classes appearing in the DGR Section 3 classification of Dangerous Goods and that they are known to be not Dangerous i.e. not restricted, furthermore the shipper confirms that the goods are in proper condition for transportation or passenger carrying aircraft (DGR para 8.1.23).

MARKS AND NUMBER OF PACKAGES	PROPER DESCRIPTION OF GOODS (Trade Names not permitted) SPECIFY EACH ARTICLE SEPARATELY	NET QUANTITY PER PACKAGE
OneBox in Dry Ice	DNA<br>Research Purpose	Micro-centrifuge tubes<br>( )

Name and Address of Shipper:<br><br>Dr. Biswajit Dubashi<br>Professor and PI<br>Department of Medical Oncology<br>JIPMER, Puducherry-605006<br>Contact No.:	Signature:<br><br>Stamp of the Shipper:<br><br>Date:
