# Supplementary material for: Molecular and Pharmacogenetic Marker Evaluation in Relation to the Toxicity and Clinical Response of Acute Lymphoblastic Leukemia Treatment in Indian Children (MPGx-INDALL): Protocol for a Prospective Observational Cohort Study
Source: JMIR Res Protoc. 2026 Mar 17;15:e79865. doi: 10.2196/79865 (PMC12994881; doi:10.2196/79865)
Supplement: Multimedia Appendix 3 [file resprot-v15-e79865-s003.pdf]

MPX-IND ALL Case Record Form Version 1.3

Annexure I: Baseline and treatment details proforma

A. **Patient details**:

Patient Initials: ____________________

Unique study ID: __________________

Age (completed years): ___________

Sex: Male / Female

Mobile Numbers: ___________

Assent taken or not taken: Yes/No

Consent version ___________

Was time allowed to take consent: Yes/No

Person taking consent ___________

Whether patient included in the study / date of inclusion: ___________

Hospital registration date ___________

Self-reported geographical location ___________

Community type Urban/Rural

Family history of other cancers___________

Date of Birth:

B. **Family details**:

Primary Caregiver: Mother/Father/Other (mention: ____________)

Type of family: (joint/nuclear); No. of members: ___; No. of siblings: (M)__ (F)_

Order of birth:

Family history of malignancy: Yes/No/Unknown

C. **History and examination:**

Antecedent hematological disorder: Yes/No/Unknown

(if yes: mention: ____________________)

Parameter	Options
Clinical Symptoms	
1. Fever	Yes/No no. of days.
2. Bleeding	Yes/No no. of days
3. Fatigue	Yes/No no. of days
4. CNS symptoms (headache/vomiting/blurred vision/altered sensorium)	Yes/No<br>Headache: no. of days<br>Vomiting: no. of days<br>Blurred vision: no. of days<br>Altered sensorium: no. of days
5. Others (mention)	Type of symptom: ___________<br>No. of days:
Physical Examination	
1. ECOG PS (16 to 18 years)	
2. Lansky Score (< 16 years)	
3. Lymphadenopathy	Yes/No<br>Palpable / non-palpable<br>Size:<br>Bulky lymph nodes:
4. Hepatomegaly	Yes/No (if yes) _____(cm BCM)<br>Beyond umbilicus: Yes/No
5. Splenomegaly	Yes/No (if yes) _____ (cm BCM)<br>Beyond umbilicus: Yes/No
6. Testes involved	Yes (Right)/ Yes (left) / Yes (both)/ No
7. Any evidence of mediastinal mass (CXR)	Yes /No<br>Measurement: ___________ (in cm)<br>Bulky mediastinal mass: Yes/No

D. Baseline laboratory parameters

**Investigations**	**Date (DD-MM-YYYY)**	**Value (NA for not available)**
**Complete blood count**		
Hemoglobin (g/dL)		
Total leucocyte count (/µL)		
• Neutrophil (%)		
• Lymphocyte (%)		
• Eosinophil (%)		
• Monocyte (%)		
• Basophil (%)		
• Blasts (%)		
• PS (% blasts)		

Platelet count (/µL)		
Any atypical cells (Yes / No) If yes: Mention %		
**Liver function tests / Kidney function tests**		
Urea (mg/dL)		
Creatinine (mg/dL)		
Calcium (mg/dL)		
Phosphorus (mg/dL)		
Sodium (mEq/L)		
Potassium (mEq/L)		
Uric acid (mg/dL)		
Total serum bilirubin (mg/dL)		
Direct serum bilirubin (mg/dL)		
Indirect serum bilirubin (mg/dL)		
Total protein (g/dL)		
Albumin (g/dL)		
Globulin (g/dL)		
SGOT / AST (IU/L) (Mention Lab ULN in brackets)		
SGPT / ALT (IU/L) ((Mention Lab ULN in brackets))		
ALP (IU/L) ((Mention Lab ULN in brackets)		
GGT (IU/L) ((Mention Lab ULN in brackets)		

Amylase (IU/L) ((Mention Lab ULN in brackets)		
Lipase (IU/L) ((Mention Lab ULN in brackets)		
LDH (IU/L) (Mention Lab ULN in brackets)		
Blood sugar: Random / Fasting		
**Viral markers**		
HBsAg (Reactive / Nonreactive)		
HCV Ab (Reactive / Non-reactive)		
HIV Ag (Reactive / Non-reactive)		

E. **Bone marrow**: Yes/No (Date___________)

Aspirate: Blast (%): ___________Bx (if available): _____________

Flowcytometry: (Circle positive ones) Date:_______________

B cell Markers: CD19, CD20, CD22, cCD22, cCD79a,

T cell markers: sCD3, cCD3, CD7, CD5, CD1a, CD4, CD8, CD2

Precursor markers: CD34, TdT, CD117

Other imp markers: CD45, CD9, CD10, HLA-DR, CD56, CD 73, CD86, CD81, CD 38, CD123, CD66c

Aberrant myeloid marker: MPO, CD11c, CD14, CD64, CD13, CD33

Final diagnosis: T-ALL / ETP-ALL / B-ALL / MPAL

Karyotyping:

Not done/ Failure / Normal / Abnormal

If abnormal:

No of chromosomes _____

Translocations (mention) __________________

T-ALL

- ☐ t(10;14)(q24;q11)
- ☐ t(7;19)(q34;p13)
- ☐ t(1;14)(p32;q11)
- ☐ t(1;7)(p32;q34)
- ☐ t(11;14)(p15;q11)
- ☐ t(11;14)(p13;q11)
- ☐ t(11;14)(p15;q11)
- ☐ t(5;14)(q35;q32)
- ☐ 7p15 translocations
- ☐ 11q23 rearrangements
- ☐ t(8;14)(q24;q11)
- ☐ t(9;14)(q34;q32)
- ☐ Other T-ALL
- ☐ Other ...

B-ALL

- ☐ t(12;21)(p13;q22)
- ☐ t(1;19)(q23;p13.1)
- ☐ t(17;19)(q22;p13)
- ☐ t(1;11)(q21;q23)
- ☐ t(4;11)(q21;q23)
- ☐ t(9;11)(p22;q23)
- ☐ t(10;11)(p12;q23)
- ☐ t(11;19)(q23;p13.3)
- ☐ t(9;22)(q34;q11.2)
- ☐ Other(s) ...

FISH: Date: __________; Done / Not done

- t(12:21): Y/N
- t(1:19): Y/N
- MLL/KMT2A rearrangement: Y/N
- t(9:22): Y/N

- **PCR Result**: Normal / Abnormal / Not done

Abnormal transcripts (Circle): BCR-ABL1 / TEL-AML1 / E2A-PBX1 / MLL-AF4 /

MLL-AF9 / MLL-ENL /E2ABA/TCF3-HLF/MLL BA/

Others: ____________________________________

F. Prognostic categorization: (ICICLE)

- Risk category at day 1: Standard Risk / Intermediate Risk / High Risk (for B-ALL)/ High Risk (T-ALL)
- Day 8 Risk category: Standard Risk / Intermediate Risk / High Risk (for B-ALL)/ High Risk (T-ALL)
- Post Induction Risk Category: Standard Risk / Intermediate Risk / High Risk (for B-ALL)/ High Risk (T-ALL)

G. CNS disease: Yes / No / Traumatic / Not done: CNS 1 / 2 / 3

H. Other relevant information:

I. Treatment Details: ICiCLe Protocol

Induction: Date of start: ________________Date of completion: _________________

(Drugs as applicable as per risk category)

**Drug**	**Planned dose / number**	**Received dose/days (Cumulative)**	**Overall dose intensity (%)**	**Remarks (record dose omissions)**
Steroid Dexamethasone				
Steroid Prednisolone				
Vincristine				
L-Asparaginase				
Daunorubicin				
Tyrosine kinase inhibitor<br><br>(Specify)				
Intrathecal methotrexate				

Induction Outcome: Date: ____________

- **Day 8 PS Blast count: ________ / Not available**
- **End of induction Bone marrow:**

  Blast (%)

  Remission status: CR / Not CR / Dilute / Not available
- **MRD Status: (%): _____   MRD Positive / Negative / Dilute / Not available**

Induction Mortality: Yes / No

If mortality: Cause: Disease / Infection / Toxicity

Consolidation: Date of start: _________________Date of completion:_________

(Drugs as applicable as per risk category)

**Drug**	**Planned dose / days**	**Received dose/days (Cumulative)**	**Overall dose intensity (%)**	**Remarks (record dose omissions)**
6-Mercaptopurine				
Cyclophosphamide				
Cytarabine				
Vincristine				
L-Asparaginase				
Intrathecal methotrexate				
TKI (Specify)				

Consolidation Outcome: Date:_________

- **End of Consolidation Bone marrow: Blast (%)**
- **Remission status: CR / Not in CR / Dilute / Not done**
- **MRD Status: (%): ____ MRD Positive / Negative / Dilute / Not done**

Radiation Details:

Site	Dose	Start (date)	End (date)
Cranial			
Testicular: Right			
Testicular: Left			

Interim Maintenance: Date of start: _______________ Date of completion: __________

Drug	Planned dose / days	Received dose/days (Cumulative)	Overall dose intensity (%)	Remarks (record dose omissions)
6-Mercaptopurine				
Methotrexate (oral)				
Methotrexate (IV) Capizzi				
HD-Methotrexate				
Vincristine				
Intrathecal Methotrexate				
TKI (specify)				

Delayed Intensification: Date of start:______________;Date of completion:__________

**Drug**	**Planned dose / days**	**Received dose/days (Cumulative)**	**Overall dose intensity (%)**	**Remarks (record dose omissions)**
Steroid Dexamethasone				
Doxorubicin				
Mitoxantrone				
6-Mercaptopurine				
Cyclophosphamide				
Cytarabine				
Vincristine				
L-Asparaginase				
Intrathecal methotrexate				
TKI (Specify)				

Maintenance ____: Date of start: ___________________________________

Date of Maintenance __ completion: _________________________________

**Drug**	**Planned dose / days**	**Received dose/days (Cumulative)**	**Overall dose intensity (%)**	**Remarks (record dose omissions)**
6-Mercaptopurine				
Methotrexate (oral)				
Intrathecal Methotrexate				
TKI (specify)				

Maintenance __ : Date of start: ___________________________________

Date of Maintenance __ completion: _________________________________

**Drug**	**Planned dose / days**	**Received dose/days (Cumulative)**	**Overall dose intensity (%)**	**Remarks (record dose omissions)**
6-Mercaptopurine				
Methotrexate (oral)				
Intrathecal Methotrexate				
TKI (specify)				

Maintenance __ : Date of start: ___________________________________

Date of Maintenance __ completion: _________________________________

**Drug**	**Planned dose / days**	**Received dose/days (Cumulative)**	**Overall dose intensity (%)**	**Remarks (record dose omissions)**
6-Mercaptopurine				
Methotrexate (oral)				
Intrathecal Methotrexate				
TKI (specify)				

Follow up details:

**Date**	**Clinical S/S Asymptomatic / Symptomatic**	**CBC**	**PS**	**Syst. exam/ testes**

Relapse details: (if any)

Date of relapse: ________________ Bone marrow / CNS / Testicular

Early / Late

Mortality: Yes / No

Date of mortality: _______________;

Time of death: On treatment (mention phase) / Off Treatment

Cause of mortality: Disease / Toxicity / Infection / Other (_________________________)

Lost to follow up: Yes / No

Date of last follow up: ____

HSCT details (if done):

Response at which transplant done: CR1 / CR2 / CR 3 / _____

MRD status pre-transplant: Positive / Negative / Not available

Type of HSCT: Allogeneic MSD / Allogeneic Haploidentical / Allogeneic MUD / Others (mention) : _______________

Date of transplantation: ______________

Conditioning regimen used:

1. TBI / Cy
2. TBI / VP-16
3. Bu / Cy

4. Flu / Bu
5. TT / Flu / Bu
6. Treo / Cy
7. Treo / Flu
8. Others: ________

Transplant related mortality: Yes / No

If yes: Cause: Infection / Drug toxicity / GVHD / Others (mention) ___________

____

Annexure II: Socio-economic data (Kuppuswamy scale: 2021)

Education of head of household: ______________________

Occupation of head of household: _____________________

Total monthly family income: ________________________

Socio-economic status: ______________________________

Table 1: Occupation of the Head of the family

S. No.	Occupation of the Head	Score
1	Legislators, Senior Officials & Managers	10
2	Professionals	9
3	Technicians and Associate Professionals	8
4	Clerks	7
5	Skilled Workers and Shop & Market Sales Workers	6
6	Skilled Agricultural & Fishery Workers	5
7	Craft & Related Trade Workers	4
8	Plant & Machine Operators and Assemblers	3
9	Elementary Occupation	2
10	Unemployed	1

Table 2: Education of the Head ofthe family

S. No.	Education of the Head	Score
1	Profession or Honours	7
2	Graduate	6
3	Intermediate or diploma	5
4	High school certificate	4
5	Middle school certificate	3
6	Primary school certificate	2
7	Illiterate	1

Table 3: Total monthly income of the family

S. No.	Updated Monthly Family Income in Rupees (2012)	Updated Monthly Family Income in Rupees (2018)	Updated Monthly Family Income in Rupees (2020)	Updated Monthly Family Income in Rupees (2021)	Score
1	≥ 30,375	≥ 126,360	≥ 199,862	≥123,322	12
2	15,188–30,374	63,182–126,359	99,931–199,861	61,663-123,321	10
3	11,362–15,187	47,266–63,181	74,755 –99,930	46129-61,662	6
4	7594–11,361	31,591–47,265	49,962–74,755	30,831-46,128	4
5	4556–7593	18,953–31,590	29,973– 49,961	18,497-30,830	3
6	1521–4555	6327–18,952	10,002–29,972	6,175-18,496	2
7	≤ 1520	≤ 6326	≤ 10,001	≤ 6174	1

Table 4: Kuppuswamy socio-economic status scale 2021

S. No.	Score	Socioeconomic Class
1	26–29	Upper (I)
2	16–25	Upper Middle (II)
3	11–15	Lower Middle (III)
4	5–10	Upper Lower (IV)
5	< 5	Lower (V)

Adapted from Sheikh Mohd Saleem et al. Indian Journal of Forensic and Community Medicine 2021

Annexure III: Toxicity recording proforma (Part A)

Toxicity event serial number	Event date (of onset)	NCI CTCAE Category	Organ system involved	Grade at onset (site-specific if applicable)	Phase / Day of treatment protocol	Date of resolution	Maximum grade (between onset & resolution)	Primary attributable cause #1/#2/#3	Attributable causes (if applicable) (including supportive care drugs)	Related toxicities (write related toxicity event numbers) **	Final outcome (Death / Resolution with deficits/Ongoing toxicity/ complete resolution) #	Remarks

Toxicity recording proforma (Part B)

Clinical investigations required: Yes/No

	If Outpatient treatment		If inpatient treatment			Infection details							
Toxicity event serial number	Outpatient supportive care (Mention what care and number of days)	Outpatient antibiotics (Name & number of days) Magnex / Amikacin / Levofloxacin /Meropenem	Number of days of hospitalization	Inpatient IV antibiotics (name & number of days) Zosyn / Colistin / Teicoplanin / Tigecycline / Aztreonam)	Inpatient supportive care (mention what care and number of days)	If clinical site of infection, mention Pneumonia / Mucositis /Abdominal / Perianal / Soft tissue	If blood culture +, mention Gram +/ - and ESBL / CRE / MRSA / VRE	If clinical fungal, mention Proven / Probable / possible	Antifungal (treatment)<br><br>Name and number of days<br><br>Amphotericin Voriconazole Caspofungin	Final category of infection:<br><br>CDI / MDI / CDI with MDI/ FUO	Transfusion support (mention product and number used)	Treatment delay (number of days)	Subsequent dose modification / dose omissions (mention drug name, and dose % or omissions)

Recording of toxicities

1. All toxicity event (clinical or asymptomatic laboratory) to be recorded with a **unique serial number** based on date of occurrence.
2. In treatment, mention according to specific serial number of the toxicity.
3. The name of toxicity should be recorded according to NCI CTCAE v5.0 categories (refer toxicity monitoring protocol for the list of toxicities that should be focused on at various phases of treatment protocol)
4. Any toxicity event which is outside the list will be assessed and recorded after discussion and will preferably be recorded and graded according to NCI CTCAE v5.0.
5. **Toxicity events may be related, and related toxicity numbers should be recorded in Column 10 Part A (for example: Toxicity event 5 is grade IV neutropenia detected in laboratory, toxicity event 6 may be febrile neutropenia related to event 5, toxicity event 7 may be colitis related to event 5 and 6 and so on; so till resolution of event 5, other related toxicities to event 5 is to be recorded in column 10)
6. # <u>For any toxicity which is ongoing > 4 week with no resolution to be documented as ongoing toxicity</u>
7. **For infective episodes, document as CDI (Clinically documented infection) or MDI (Microbiologically documented infection) or CDI+MDI or FUO (fever of unknown origin); for MDI: Mention organism categorized as above.**
8. Important medications for supportive care are to be recorded (especially need for anticoagulants, anti-epileptics).
9. Frequency of clinical and laboratory monitoring (Follow toxicity monitoring protocol: Annexure IV).
10. Primary attributable cause: The most proximate attributable cause for that toxicity as decided by the clinician to be mentioned;
11. Other attributable causes: To mention all causes which may be attributed to that particular toxicity but may not be most proximate. (Follow chart)

Attributable causes of toxicity: Broad categories + record any other attributable cause under others

Primary disease	Chemotherapeutic drugs	Supportive antimicrobials	Therapeutic antimicrobials	Supportive medications / measures	Infection related	Treatment related
• Baseline disease<br>• Disease (relapse/refractory)	• Steroid (prednisolone/ dexamethasone)<br>• Vincristine<br>• L-Asparaginase<br>• Daunorubicin / Doxorubicin<br>• Mitoxantrone<br>• Cyclophosphamide<br>• Cytarabine<br>• 6MP<br>• Methotrexate (high dose)<br>• Methotrexate (low dose)<br>• Imatinib<br>• Dasatinib<br>• Nilotinib	• Cotrimoxazole<br>• Acyclovir<br>• Fluconazole<br>• Voriconazole<br>• Itraconazole	• Cefoperazone-sulbactam (Magnex)<br>• Amikacin<br>• Levofloxacin<br>• Meropenem<br>• Piperacillin-tazobactam (Zosyn)<br>• Imipenem<br>• Colistin<br>• Tigecycline<br>• Aztreonam<br>• Teicoplanin<br>• Vancomycin<br>• Amphotericin B<br>• Caspofungin<br>• Cotrimoxazole<br>• Acyclovir<br>• Fluconazole<br>• Voriconazole<br>• Itraconazole<br>• Posaconazole	• GCSF<br>• Ondansetron<br>• Granisetron<br>• Aprepitant<br>• Fosaprepitant<br>• PRBC transfusion<br>• Platelet transfusion<br>• Granulocyte transfusion<br>• Leucovorin<br>• NaHCO3	• Sepsis<br>• Viral Hepatitis<br>• CNS infection<br>• Head and neck infection<br>• Oral cavity infection<br>• Thoracic infection (Pneumonia)<br>• Skin and soft tissue infection<br>• Abdominal infection (cholangitis / enterocolitis)<br>• Perianal infection<br>• Genitourinary infection	• Injection related<br>• Central line related

Annexure IV: Toxicity monitoring protocol (NCI CTCAE v 5.0 to be followed)

1. **Common minimum frequency of clinical/laboratory monitoring:**
   - Induction: Weekly once (Physical)
   - Consolidation/Interim Maintenance/Delayed Intensification: Once in two weeks (Physical)
   - Maintenance (1st 100 days): Once in two weeks (Physical/telephonic)

2. **Clinical symptom assessment list at each physical visit (By project staff)**
   1. To assess general status: Document encephalopathy or delirious state if any. **(resident doctor to do)**
   2. Document any clinical complaints (Grade according to CTCAE v5.0 if already in list below) else document the complaint verbatim.
   3. Follow the checklist to document any toxicity if any (To be done by research Nurse). If any toxicity, proceed to Annexure III to document the details regarding the toxicity.
   4. Ask and grade if any cough/dyspnea. **(not in maintenance)**
   5. Ask and grade nausea, vomiting, abdominal pain, diarrhea, constipation.
   6. Ask and grade symptoms of peripheral sensory and motor neuropathy.
   7. Ask and grade if any allergic reaction/anaphylaxis in medications received since last visit.
   8. Ask/Examine and grade infusion site extravasation (if any) **(not in maintenance)**
   9. Ask/examine and grade mucositis.
   10. Weight in each visit (Baseline Weight/height/MUAC/Waist circumference/Hip circumference)
   11. BP measurement in each visit **(Induction/consolidation only)** and grade according to pediatric age specific BP charts
   12. Document if any steroid stigmata and grade Cushingoid habitus **(Induction/ consolidation only)**

3. **Sample to be sent at each visit: (during maintenance phase patient may get it done by themselves)**
   1. Complete blood count
   2. Fasting blood sugar (Not in maintenance)
   3. Amylase and Lipase (not in maintenance)
   4. Liver function test, Kidney function test including serum electrolytes and magnesium.

**For clinical suspicion or patient complaints or every case of FN:

Following sites of infection should be looked for and graded separately: (**to be recorded by project staff after examination by a resident doctor**)

Skin/soft tissue infection, Lung infection or pneumonia, Colitis, Anorectal infection, Mucositis, Oral thrush.

Rest sites: Document as per presentation/clinical diagnosis

For fungal infection: Document as possible/probable/Proven.

4. **Clinical investigations in special scenarios:**
    1. Febrile neutropenia: Blood culture, CXR (minimum)
    2. Febrile neutropenia not responding to first line antibiotics: Serum procalcitonin, Serum galactomannan.
    3. Clinical suspicion of colitis: USG Abdomen
    4. Bleeding events / Sepsis: DIC profile (twice weekly) (Induction/consolidation)
    5. Clinical Pneumonia / FN not responding to antibiotics for 96 hours: HRCT Chest.
    6. Grade 3 and 4 Transaminases: Viral markers
    7. Clinical suspicion of pancreatitis: USG abdomen or CT abdomen (if USG non-contributory)
    8. Clinical suspicion of DVT / CVT: USG doppler or CECT Brain

**Organ System**	**Toxicity**	**Phase of Treatment when to monitor for toxicity (ICiCLe Protocol)**					**Remarks**
		**Inductio n**	**Consolidatio n**	**Interim Maintenan ce**	**Delayed intensificatio n**	**Maintenan ce**	
Hematological	Anemia	Y	Y	Y	Y	Y	1. **Capture only grade 3 or 4.**<br>2. Minimum Monitoring weekly during induction,

							once in two weeks during rest of protocol.<br>**3. Capture number of PRBC transfusion at each event**
	Neutropenia	Y	Y	Y	Y	Y	**1. Capture only grade 3 or 4.**<br>2. Minimum Monitoring weekly during induction, once in two weeks during rest of protocol.<br>**3. Capture if GCSF used and if used then, days of GCSF used at each event**
	Thrombocytopenia	Y	Y	Y	Y	Y	**1. Capture only grade 3 or 4.**<br>2. Monitoring weekly during induction, once in two weeks during rest of protocol.<br>**3. Capture and grade purpura**<br>**4. Other bleed events capture separately (Grade)**

							5. **Capture number of SDP/RDP used at each event.**
	Bleeding event (based on site)	Y	Y	Y	Y	Y	1. **Document all events and record grade site-specific**<br>2. All patients to be asked/monitored at every event of Grade 3 or 4 thrombocytopenia/DIC/ critical sickness
	Febrile neutropenia	Y	Y	Y	Y	Y	1. Document each event
	DIC	Y	Y	--	--	--	1. **Capture only grade 3 and 4.**<br>2. Laboratory evaluation to be done only in bleeding events or critical sickness Febrile neutropenia (twice weekly)

Vascular	Thromboembolic events	Y	Y (HR)	--	Y	--	1. Record thromboembolic event as per CTCAE (site to be documented)<br>2. Grade 2 and above to captured.<br>3. Imaging studies only if clinically indicated
	Hypertension	Y	Y	--	--	--	1. Frequency of Minimum Monitoring weekly during induction, once in two weeks during rest of protocol.<br>2. **Pediatric BP charts** and to record grade accordingly
Cardiac	Heart failure	Y	Y	Y	Y	--	
Endocrine	Adrenal insufficiency	Y	Y	--	--	--	1. To capture grade 3 and above<br>2. Evaluation only if clinically indicated
	Cushingoid	Y	Y	--	--	--	1. Clinical grading grade 3 or above
	Hyperglycemia	Y	Y (HR)	Y	Y	--	1. FBS may be done at each sampling and based on intervention, grading

Gastro-intestinal	Abdomen pain	Y	Y	Y	Y	Y	1. To ask at each visit
	Constipation	Y	Y	Y	Y	Y	1. To ask in each visit
	Diarrhea	Y	Y	Y	Y	Y	1. To ask in each visit
	Gastritis	Y	Y	Y	Y	Y	1. To ask in each visit
	Oral mucositis	Y	Y	Y	Y	Y	1. To ask and examine at each visit
	Nausea	Y	Y	Y	Y	Y	1. To ask and grade at each visit
	Vomiting	Y	Y	Y	Y	Y	1. To ask and grade at each visit
Hepato pancreatico biliary	Pancreatitis	Y	Y (HR)	--	Y	--	To capture all grades in conjunction with serum amylase/lipase (imaging study only if indicated)
	ALT increased	Y	Y	Y	Y	Y	**1. Capture only grade 3 or 4.**<br>2. Minimum Monitoring weekly during induction, once in two weeks during rest of protocol.<br>1.
	ALP increased	Y	Y	Y	Y	Y	**1. Capture only grade 3 or 4.**<br>2. Minimum Monitoring weekly during induction, once in two weeks

							during rest of protocol.
	AST increased	Y	Y	Y	Y	Y	1. **Capture only grade 3 or 4.**<br>2. Minimum Monitoring weekly during induction, once in two weeks during rest of protocol.
	Hypoalbuminem ia	Y	Y	Y	Y	Y	1. **Capture only grade 3 or 4.**<br>2. Minimum Monitoring weekly during induction, once in two weeks during rest of protocol.
	Blood bilirubin increased	Y	Y	Y	Y	Y	1. **Capture only grade 3 or 4.**<br>2. Minimum Monitoring weekly during induction, once in two weeks during rest of protocol.
	Serum amylase increased	Y	Y (HR)	--	Y	--	1. Capture all grades.

							2. Minimum Monitoring weekly during induction, once in two weeks during rest of protocol.
	Lipase increased	Y	Y (HR)	--	Y	--	1. Capture all grades.<br>2. Minimum Monitoring weekly during induction, once in two weeks during rest of protocol.
General disorders and administration site conditions	Fever	Y	Y	Y	Y	Y	Document all events with temperature grading as reported by patient
	Infusion site extravasation	Y	Y	Y	Y	Y	Capture and grade all events
Immune system disorders	Allergic reaction	Y	Y	Y	Y	Y	Capture and grade all events
	Anaphylaxis	Y	Y	Y	Y	Y	Capture all events
Infections	Viral Hepatitis	Y	Y	Y	Y	Y	Document and grade each event<br>**Viral markers to be sent at each event of grade 3 or 4 transaminases**
	CNS infection (meningitis / encephalitis	Y	Y	Y	Y	Y	Capture and grade all events

	Head and neck infection (periorbital / Eye / ear infection Rhino- sinusitis) Specify site	Y	Y	Y	Y	Y	Capture and grade all events
	Oral cavity infection (tooth infection / Oral thrush) mention	Y	Y	Y	Y	Y	Capture and grade all events
	Thoracic infection (Pneumonia)	Y	Y	Y	Y	Y	1. CXR to be done in FN with any respiratory complaints.<br>2. Document and grade each events
	Skin and soft tissue infection	Y	Y	Y	Y	Y	Capture and grade all events
	Abdominal infection (cholangitis / enterocolitis)	Y	Y	Y	Y	Y	Capture and grade all events
	Perianal infection	Y	Y	Y	Y	Y	Capture and grade all events
	Genitourinary infection	Y	Y	Y	Y	Y	Document all events
Musculoskeletal and connective disorders	Generalized muscle weakness	Y	Y	--	--	--	Document if patient complains
	Avascular necrosis	Y	Y	--	--	--	Document as per event
	Headache	Y	Y	Y	Y	Y	Ask in all visits

Nervous system disorders	Intracranial Haemorhage	Y	Y	Y	Y	Y	Document all events. Evaluation as per clinical suspicion
	Seizure	Y	Y	Y	Y	Y	Document all events
	Peripheral sensory neuropathy	Y	Y	Y	Y		Ask and assess in each visit
	Peripheral motor neuropathy	Y	Y	Y	Y		Ask and assess in each visit
Renal and urinary disorders	Creatinine increased	Y	Y	Y	Y	Y	1. Capture all grades.<br>2. Minimum Monitoring weekly during induction, once in two weeks during rest of protocol.
	Hyperkalemia	Y	Y	Y	Y	Y	1. Capture all grades.<br>2. Minimum Monitoring weekly during induction, once in two weeks during rest of protocol.
	Hyperuricemia	Y	Y	Y	Y	Y	1. Capture all grades.<br>2. Minimum Monitoring weekly during induction, once in two weeks

							during rest of protocol.
	Hyponatremia	Y	Y	Y	Y	Y	1. Capture all grades.<br>2. Minimum Monitoring weekly during induction, once in two weeks during rest of protocol.
	Hypoalbuminemia	Y	Y	Y	Y	Y	Remove<br>Already in hepato-pancreaticbiliary
	Hypokalemia	Y	Y	Y	Y	Y	1. Capture all grades.<br>2. Minimum Monitoring weekly during induction, once in two weeks during rest of protocol.
	Hypomagnesemia	Y	Y	Y	Y	Y	1. Capture all grades.<br>2. Minimum Monitoring weekly during induction, once in two weeks during rest of protocol.
	Tumor lysis syndrome	Y	--	--	--	--	To document in every case till 1 week of induction

Respiratory,thoracic and mediastinal disorders	Dyspnea	Y	Y	Y	Y	--	Ask and grade each visit
	Hypoxia	Y	Y	Y	Y	--	1. To document only in critical illness/ Pneumonia
	Cough	Y	Y	Y	Y	--	2. Ask and grade each visit
	Pleural effusion	Y	Y	Y	Y	Y	1. Document as per presentation.
Skin	Rash	Y	Y	Y	Y	Y	**1.** Characterize all rash (Maculo-papular / purpuric / vesiculo-bullous) **(resident doctor to do)**<br>2. Examine and grade at presentation.<br>3. Purpura to be documented separately

Toxicity checklist (Tick for yes / X for No) at each clinic visit.

**Date**<br>**Chemo phase / day (eg. 01.01.2023, Ind D9)**								
Toxicity								
Blood pressure								

Hypertension								
Febrile Neutropenia								
CNS Infections (Meningitis/encephalitis)								
Thoracic infection (Pnemonia)								
Abdominal Infection (cholangitis/entercoities)								
Perineal infections								
Head and Neck infections (Periorbital/Eye/ear infection/ rhino sinusitis)								
Oral Cavity infection (Tooth/Oral)								
Skin and Soft tissue infection								
Genitourinary infection								
Abdominal Pain								
Constipation								
Diarrhea								
Gastritis								
Oral Mucositis								
Nausea								
Vomiting								
Fever								

Infusion site extravasation								
Allergic reactions								
Anaphylaxis								
Generalized muscle weakness								
Headache								
Peripheral Sensory neuropathy								
Peripheral neuropathy								
Seizure								
Dyspnea								
Hypoxia (only if dyspnea check SpO2)								
Cough								
Skin rash								
Avascular necrosis								
Any bleeding								
CNS hemorrhage								

Annexure V: Follow up investigation record proforma

**Date**	**Normal**								
**Hemoglobin**									
**Platelet count**									
**TLC**									
**DLC**									

**ANC**									
**Urea**									
**Creatinine**									
**Calcium**									
**Phosphate**									
**Uric acid**									
**Sodium**									
**Potassium**									
**Total bilirubin**									
**Direct**									
**Indirect**									
**Total protein**									
**Albumin**									
**Globulin**									
**SGOT**									
**SGPT**									
**ALP**									
**Amylase**									
**Lipase**									
**Magnesium**									
**Viral markers**									

Annexure VI: Anthropometric and Blood pressure record proforma

SOP for anthropometry: MUAC is to be measured in left arm only. Anthropometric measurement interpretation: Use Anthrocal app (Developed in AIIMS).

Height for age, weight for age and weight for height: WHO standards for <5 years of age, IAP standards for ≥ 5 years.

For BMI, MUAC, HC: Follow WHO standards

Baseline: Weight (Kg): ____ Height (cm): _____; Head Circumference(cm): _______; MUAC (cm): ______;

Waist circumference (cm): _______ ; Hip Circumference: _________

BP: ____/_____; BMI: ______ ; BSA: _______

Height for Age: _______ Weight for Age: _______ BMI for Age: _______ MUAC for Age: _______

**Date**										
**Pusle rate**										
**BP**	**Systolic**									
	**Diastolic**									
**Weight**										
**Height (3 monthly)**										

**Date**										
**Pulse rate**										
**BP**	**Systolic**									
	**Diastolic**									
**Weight**										
**Height (3 monthly)**										

Annexure VI: Sample Log

**Sample type<br><br>(circle A or B whichever applicable)**	**Sam ple code**	**Collected Yes / No If No (mention reason)**	**Date of collection**	**Date of process ing**	**DNA Quant ity (ng/ul)**	**A260/2 80**	**A280/2 30**	**Qubit readin g (ng/ul)**	**Tapestat ion DIN**	**Date of shippin g**	**Bioban ked (tick if yes)**	**Remark (mentio n if sample not analysa**

												**ble/ stored with reason)**
**BASELINE**												
**1. Baseline germline sample**<br>**A. Buccal swab**<br>**B. Saliva**												
**2. Baseline onco-genome sample**<br>**A. Bone marrow**<br>**B. Peripheral Blood (Blasts >80%)**												
**3. Baseline plasma for proteomics**					**--**	**--**	**--**	**--**	**--**			
**REMISSION (AT END OF INDUCTION)**												
**1. Post remission**												

**germline sample 1**<br>**A. Saliva**<br>**B. Buccal swab**												
**2. Post remission germline sample 2**<br>**A. Peripheral blood (MRD Negative)**												
**3. Post remission plasma sample for proteomics**					--	--	--	--				
**DRUG LEVELS**												
**Drug name**	**Sample code**	**Date of drug administration of drug**	**Time of drug administration**	**Day / Phase of protocol**	**Drug brand name**	**Date of sample collection**	**Time of sample collection**	**Time difference (hours)**	**Date of sample processing**	**Time of sample processing**	**Date of analysis**	**Remarks (if hemolysed / not anlysed)**
**1. Vincristine**												
**2. L-asparaginase**												
**3. High-dose methotrexate**												
**4. 6-MP**												

**5. Low-dose methotrexate**												
**6.**												
**7.**												

Annexure VII: PedsQL (Version / Language as applicable)

1. PedsQL V3.0 Cancer Module Parent Report (13-18), Child Report (13-18),

2. PedsQL V3.0 Cancer Module Parent Report (8-12), Child Report (8-12),

3. PedsQL V3.0 Cancer Module Parent Report (5-7), Child Report (5-7),

4. PedsQL V3.0 Cancer Module Parent Report (2-4), Child Report (2-4),

Annexure VIII: Nutrition proforma: To be filled by the nutritionists.

NUTRITIONAL ASSESSMENT

1. **Hospital Number:** **2. Study ID:** **3. IRCH:**
4. **Chemotherapy Phase-** **5. Day of ICICLE Protocol Phase:**
6. **NATAL HISTORY**

Normal /LSCS

Birth weight –______Normal/ LBW/ VLBW/ ELBW

7. **CLINICAL EVALUATION / CLINICAL SIGNS**

	ACCEPTABLE	MALNUTRITION	OTHER POSSIBLE CAUSES
	• clear pink membranes	• Dryness	• allergies
LIPS	• Smooth	• Dry, cracked<br>• Sore corners of lips	• Sunburn, windburn
MOUTH AND GUMS	• Red tongue without swelling<br>• Teeth without carries<br>• Gums without swelling or bleeding	• Smooth or magenta tongue<br>• Decreased taste sensitization<br>• Swollen and bleeding gums	• Medications<br>• Poor oral hygiene
SKIN	• Smooth, firm and good colour	• Poor wound healing<br>• Dry and rough<br>• Lack of fat under skin<br>• Bruising and bleeding under skin	• Poor skin care<br>• Diabetes mellitus<br>• Ageing<br>• Medications
NAILS	• Smooth firm pink	• Ridged nails<br>• Spoon shaped nails<br>• Pale nails	
OTHERS		• Dementia<br>• Swollen glands at front of neck<br>• Bowed legs	• Disorders of ageing<br>• Diabetes mellitus
HAIR	• Shiny<br>• Firm scalp	• Dull,brittle,dry<br>• Easy pluckibility<br>• Flag sign, copper hair	• Therapy induced loss of hair
EYES	• Bright	• Spots<br>• Redness at corners of eyes	• Anaemia<br>• Eye disorders
	• clear pink membranes	• Dryness	• allergies

8. **DIET HISTORY**

a. **Exclusive breast feeding done for how many months –**

b. **Currently on BF/ Complementary foods/ Both –**

c. **Started weaning at the month of –**

d. **Schedule feeding / Demand feeding**

e. **If on complementary foods**

   i. **Mixed/ Veg /Ova- veg**

   ii. **Diet type – liquid/ semi liquid/semi solid / soft solid/solid**

   iii. **Meal timing-**

   iv. **Food frequency-**

   v. **Food allergy-**

   vi. **Supplements if any taken-**

9. **NUTRIENT COUNT /24 HRS RECALL**

NUTRIRNTS	a.INTAKE	b.REQUIREMENT	c.MET%	d.DEF%
ENERGY(k.calc)				
PROTEIN (gm)				

11. PLAN / RECOMMENDATION

a. **TYPE OF DIET- ORAL/ENTERAL/PARENTERAL**

b. **ENERGY (k.cal/d)**

c. **CHO (gms/d) –**

d PROTEIN(gms/ d)

e. FAT(gms/d) -

f. FLUID Intake (ml/d) -
