# Supplementary material for: Molecular and Pharmacogenetic Marker Evaluation in Relation to the Toxicity and Clinical Response of Acute Lymphoblastic Leukemia Treatment in Indian Children (MPGx-INDALL): Protocol for a Prospective Observational Cohort Study
Source: JMIR Res Protoc. 2026 Mar 17;15:e79865. doi: 10.2196/79865 (PMC12994881; doi:10.2196/79865)
Supplement: Multimedia Appendix 5 [file resprot-v15-e79865-s005.docx]

**Objective 2**. This is an explorative objective; hence, the top hit variants will be studied in the rest of the cohort. However, with n = 250, a power calculation simulation showed a power of β = 0.70 with an α of 0.05, using the power GWAS interaction tool (187). This was done using the tool’s variant and environment interaction mode, where the environment’s effect (OR: 4.5) is enhanced by the variant (combined OR: 10.0). This power calculation assumed 5000 filtered markers for association analysis, with minor allele frequencies of 0.4, and an environment (drug) exposure of 0.4. Multiple comparisons corrected the overall significance level for interaction testing, set at 0.05 in a case-only analysis, with the environment and variants as independent factors. Based on the assumptions mentioned above, we will thus have sufficient power for a combined analysis including only the top 100 candidates.

**Objective 3**. This objective is for the biobanking of biological and clinical material and will serve as the appropriate size for searching for any new biomarkers in this treatment setting. e.g. for plasma marker association analysis, a sample size of 172 samples was calculated per arm for an effect size of 0.43 to reach a power of 80%, if one group differs from the overall mean by one standard deviation (0.5), Ninety-two biomarkers would be measured from each sample. A group of 100 healthy volunteers from a similar age group will be used to examine the relative differences
